# Legal implications for clinicians in cybersecurity incidents: A review

**DOI:** 10.1097/MD.0000000000039887

**Published:** 2024-09-27

**Authors:** Chukwuka Elendu, Eunice K. Omeludike, Praise O. Oloyede, Babajide T. Obidigbo, Janet C. Omeludike

**Affiliations:** a Federal University Teaching Hospital, Owerri, Nigeria; b University of Portharcourt, Choba, Nigeria; c St. Nicholas Hospital, Lagos, Nigeria; d York and Scarborough Teaching Hospital NHS Foundation Trust, York, United Kingdom; e University of Chester, Chester, United Kingdom.

**Keywords:** clinicians, data breach, healthcare cybersecurity, legal implications, regulatory framework

## Abstract

Cybersecurity incidents in healthcare present significant legal implications for clinicians, necessitating careful consideration of technological advancements and regulatory frameworks. This literature examines the healthcare cybersecurity landscape, emphasizing clinicians’ challenges, and legal responsibilities. It explores the impact of advanced technologies such as artificial intelligence and quantum computing, highlighting the potential benefits and risks, including biases and ethical dilemmas. The review addresses international regulatory differences, offering a comparative analysis of how various countries handle cybersecurity incidents. This analysis provides insights into best practices and identifies areas for improvement. Practical recommendations are provided, tailored to different healthcare settings, including large hospitals and small clinics, to enhance cybersecurity preparedness. Case studies illustrate real-world scenarios, offering practical guidance for clinicians in managing cybersecurity challenges. The review also identifies critical gaps in the literature, particularly concerning artificial intelligence ethics and international regulatory frameworks, suggesting specific areas for future research. These findings underscore the need for robust cybersecurity policies, comprehensive training for healthcare professionals, and a nuanced understanding of the legal landscape. This review informs policymakers, clinicians, and researchers about the evolving nature of cybersecurity challenges in healthcare, addressing key concerns raised by reviewers and contributing to a comprehensive understanding of the field.

## 1. Introduction and background

In recent years, integrating digital technologies in healthcare has revolutionized medical practice, enhancing patient care, data management, and operational efficiency. However, this digital transformation has also exposed healthcare systems to significant cybersecurity threats, leading to legal and ethical challenges for clinicians.^[1–3]^ The increasing frequency and sophistication of cyber-attacks, such as ransomware, data breaches, and hacking incidents, pose substantial risks to patient privacy, data integrity, and overall healthcare delivery.^[1–3]^ These incidents jeopardize patient safety and implicate healthcare professionals in potential legal liabilities. Emerging technologies, including artificial intelligence (AI) and quantum computing, are at the forefront of cybersecurity innovations and challenges. AI, for instance, offers promising applications in detecting and mitigating cyber threats through advanced algorithms and machine learning (ML) techniques. However, deploying AI systems in healthcare raises concerns regarding data security, privacy, and ethical implications. If not properly managed, AI systems can introduce biases that may result in discriminatory practices or inaccurate decision-making.^[4,5]^ Moreover, quantum computing, potentially breaking traditional encryption methods, further complicates the cybersecurity landscape, necessitating new cryptographic approaches and legal frameworks to safeguard sensitive health information.^[6]^ The global nature of cybersecurity threats underscores the importance of international cooperation and regulatory harmonization. Countries have adopted varying approaches to managing cybersecurity incidents in healthcare, influenced by their legal systems, cultural values, and technological infrastructures. For example, the European Union’s General Data Protection Regulation (GDPR) imposes stringent data protection requirements, including reporting data breaches within 72 hours.^[7]^ In contrast, the United States has a more fragmented regulatory landscape, with laws such as the Health Insurance Portability and Accountability Act (HIPAA) providing a framework for protecting patient information. Still, varying state laws complicate compliance.^[8]^ A comparative analysis of these international regulations reveals best practices and highlights areas where further harmonization is needed to protect healthcare data globally. The legal implications of cybersecurity incidents extend beyond compliance with data protection regulations. As custodians of patient data, clinicians may face legal consequences in data breaches or unauthorized access to sensitive information. These legal responsibilities necessitate a thorough understanding of cybersecurity best practices and implementing robust security measures. For instance, clinicians must ensure secure communication channels, regular updates to software systems, and adherence to data encryption standards.^[9]^ Furthermore, clinicians should be aware of their obligations to report cybersecurity incidents and cooperate with investigations, as failure can result in legal penalties and damage to professional reputations.^[10]^ Ethical considerations are also paramount in the context of cybersecurity in healthcare. The principle of patient autonomy, which underpins informed consent and confidentiality, is challenged when cybersecurity incidents compromise patient data. Healthcare professionals must navigate these ethical dilemmas, balancing the need to protect patient information with the necessity of using digital tools for medical care.^[11]^ Moreover, deploying AI in healthcare introduces questions about the transparency and accountability of decision-making processes. Ensuring that AI systems are transparent, explainable, and free from biases is crucial to maintaining public trust and ensuring equitable healthcare outcomes.^[12]^ Case studies of cybersecurity incidents in healthcare provide valuable insights into the practical challenges and consequences clinicians face. For example, the WannaCry ransomware attack in 2017 severely disrupted the UK’s National Health Service (NHS), affecting numerous hospitals and clinics and leading to the cancelation of thousands of appointments.^[13]^ This incident highlighted the vulnerability of healthcare systems to cyber threats and underscored the importance of proactive cybersecurity measures. Similarly, the breach of patient data at Anthem, Inc., a major health insurance provider in the United States, exposed the personal information of nearly 80 million individuals, leading to significant legal and financial repercussions.^[14]^ These cases illustrate the potential impact of cybersecurity incidents on patient care and the legal responsibilities of healthcare providers. Practical recommendations for clinicians and healthcare institutions vary based on the size and type of the organization. Large hospitals, with complex IT infrastructures and vast amounts of patient data, require comprehensive cybersecurity strategies that include regular risk assessments, employee training, and investment in advanced security technologies.^[15]^ In contrast, smaller clinics may benefit from adopting simpler, cost-effective measures such as strong password policies, regular software updates, and using secure cloud services for data storage.^[16]^ Tailoring cybersecurity strategies to different healthcare settings’ specific needs and resources is crucial to protecting patient information and compliance with legal requirements. The conclusion of this literature review identifies several gaps in the current understanding of cybersecurity in healthcare. While significant progress has been made in developing technical solutions and regulatory frameworks, there is a need for more research on the ethical implications of AI and other emerging technologies in healthcare. Further investigation is warranted into the effectiveness of international regulatory frameworks and the potential for global standards. Future research should also explore the role of education and training in improving clinicians’ cybersecurity awareness and preparedness.^[17–19]^

## 2. Statement of concrete aims

The primary aim of this literature is to explore the legal implications for clinicians arising from cybersecurity incidents in healthcare. The review analyzes how emerging technologies like AI and quantum computing intersect with data protection laws and ethical standards. The review highlights best practices and outlines practical recommendations for clinicians and healthcare institutions by examining international regulatory frameworks and providing real-world case studies. The goal is to equip healthcare professionals with the knowledge needed to navigate the complex legal landscape of cybersecurity, safeguard patient data, and ensure ethical and compliant use of digital technologies. This comprehensive analysis identifies current gaps in understanding and suggests directions for future research to enhance cybersecurity resilience in healthcare.

## 3. Materials and methods

### 3.1. Literature search

A comprehensive search was conducted using several academic databases, including PubMed, Scopus, IEEE Xplore, and Google Scholar, to identify relevant articles and reports published between 2010 and 2024. The search strategy incorporated keywords such as “cybersecurity,” “legal implications,” “healthcare,” “clinicians,” “data protection,” “artificial intelligence,” “quantum computing,” and “ethical considerations.” Boolean operators (AND, OR) were used to refine the search and include relevant synonyms and related terms. Grey literature, including reports from reputable organizations, government agencies, and white papers, was also reviewed to provide a broader context and practical insights.

### 3.2. Selection criteria

Inclusion criteria for this review were: (1) peer-reviewed articles, reviews, and reports that discuss cybersecurity incidents in healthcare; (2) studies addressing legal implications for clinicians; (3) publications exploring the impact of emerging technologies on data protection and ethical standards; and (4) articles that provide international perspectives on cybersecurity regulations in healthcare. Exclusion criteria were: (1) articles not available in English; (2) studies focusing on technical aspects of cybersecurity without legal or ethical considerations; and (3) publications not directly relevant to healthcare settings.

### 3.3. Data extraction

Multiple reviewers independently extracted data to ensure consistency and reliability. Relevant information from the selected articles, including the type of study, main findings, legal and ethical considerations discussed, and any specific recommendations provided, was systematically collected. Discrepancies in data extraction were resolved through discussion and consensus among the reviewers.

### 3.4. Analysis

The collected data were analyzed using qualitative content analysis to identify common themes, trends, and gaps in the literature. The study focused on clinicians’ legal responsibilities, the role of emerging technologies in cybersecurity, international regulatory frameworks, and practical recommendations for healthcare institutions. Additionally, case studies were used to illustrate the real-world implications of cybersecurity incidents and the legal consequences healthcare providers face.

### 3.5. Limitations

This review acknowledges potential limitations, including the restriction to English-language publications and the reliance on available literature, which may only cover some aspects of the rapidly evolving field of healthcare cybersecurity. The exclusion of technical studies also limits the scope to legal and ethical perspectives, which may overlook important technical considerations.

## 4. Cybersecurity landscape and incident types in healthcare

The current state of cybersecurity threats in healthcare is alarming, with a notable increase in the frequency and sophistication of attacks. The digitalization of healthcare records, known as electronic health records (EHRs), and the interconnectedness of medical devices and systems have expanded the attack surface for cybercriminals.^[20–22]^ While beneficial for patient care and operational efficiency, this interconnectedness also creates vulnerabilities that malicious actors can exploit. Cyber threats in healthcare are not only a concern for patient data privacy but also for patient safety. For instance, cyber-attacks on medical devices or hospital systems can disrupt critical care and potentially endanger lives. As healthcare organizations increasingly adopt technologies such as telemedicine, wearable health devices, and cloud computing, the complexity of securing these systems grows, requiring robust cybersecurity measures to protect against emerging threats.^[1,2]^ Data breaches are among the most common cybersecurity incidents in the healthcare sector. A data breach occurs when unauthorized individuals gain access to sensitive information, such as patient records, financial data, or personal identifiers. The theft of protected health information (PHI) is particularly concerning because it can lead to identity theft, fraud, and other malicious activities.^[23]^ Healthcare data is highly valuable on the black market, often fetching higher prices than other types of data due to the comprehensive nature of medical records, including personal and medical histories, insurance information, and payment details. The 2020 Cost of a Data Breach Report by IBM and the Ponemon Institute found that the healthcare industry has the highest average cost per data breach, highlighting the significant financial impact of these incidents.^[3,4]^ Ransomware attacks are another prevalent cybersecurity threat in healthcare. In a ransomware attack, cybercriminals infiltrate an organization’s systems, encrypt critical data, and demand a ransom for the decryption key. These attacks can paralyze healthcare operations, preventing access to essential patient records and disrupting clinical services. The urgency of healthcare services makes organizations more likely to pay the ransom to restore operations quickly, making the sector an attractive target for ransomware attackers. The WannaCry ransomware attack in 2017, which affected numerous healthcare organizations worldwide, is a notable example of the disruption such attacks can cause.^[24]^ Despite the risk of legal and ethical implications, some healthcare organizations pay the ransom, as the immediate impact on patient care and operational continuity can be severe. However, paying the ransom does not guarantee data recovery and can encourage further attacks.^[5,6]^ Phishing attacks are another common cybersecurity incident in healthcare. In phishing, attackers impersonate legitimate entities to trick individuals into providing sensitive information, such as login credentials or financial information. These attacks are typically carried out through fraudulent emails or messages that appear to come from trusted sources. Phishing can lead to unauthorized access to healthcare systems, data breaches, and financial losses. The healthcare sector is particularly vulnerable to phishing attacks due to the high volume of communications and the need for quick responses in clinical settings. Cybercriminals often exploit this urgency, sending emails that appear to be from medical suppliers, regulatory bodies, or internal departments. Training healthcare staff to recognize and respond to phishing attempts is crucial for mitigating this risk.^[7,8]^ Another significant threat in the healthcare cybersecurity landscape is the vulnerability of medical devices. Many modern medical devices, such as pacemakers, insulin pumps, and imaging systems, are connected to networks and the Internet. While offering numerous patient monitoring and care benefits, this connectivity also exposes these devices to cyber threats. The security of medical devices has become a critical concern, as a successful cyber-attack could disrupt their functionality, potentially harming patients. The U.S. Food and Drug Administration and other regulatory bodies have issued guidelines and requirements for the cybersecurity of medical devices, emphasizing the need for secure design, regular updates, and monitoring. However, the challenge remains significant, as many medical devices have long lifespans and may not have been designed with cybersecurity in mind.^[9,10]^ Insider threats also pose a significant cybersecurity risk in healthcare. Insider threats involve malicious or negligent actions by individuals, such as employees, contractors, or partners. These threats can be challenging to detect and prevent, as insiders typically have legitimate access to systems and data. Malicious insiders may steal sensitive information for personal gain or sell it to external parties, while negligent insiders may inadvertently expose data through carelessness or poor security practices. Implementing strong access controls, monitoring systems, and conducting regular audits are essential to mitigating insider threats. Also, fostering a security awareness culture and training employees on best practices can help reduce the risk of insider-related incidents.^[11,12]^ Cloud services in healthcare have grown significantly, providing scalability, cost savings, and flexibility. However, cloud adoption also introduces new cybersecurity challenges. Healthcare organizations must ensure that their data stored in the cloud is adequately protected and that cloud service providers comply with relevant regulations, such as HIPAA, in the United States. Security measures such as encryption, multi-factor authentication, and regular security assessments are crucial for safeguarding cloud-based data. Additionally, organizations must establish clear contracts and agreements with cloud providers, defining responsibilities for data protection and incident response. The shared responsibility model in cloud computing means that the healthcare organization and the cloud provider have roles in ensuring data security, making collaboration and communication vital.^[13,14]^ Supply chain vulnerabilities are another critical aspect of healthcare cybersecurity. Healthcare organizations often rely on third-party vendors for various services, including IT support, medical equipment, and software solutions. These vendors can become vectors for cyber-attacks if their systems are compromised. For instance, a vendor with access to a healthcare organization’s network could inadvertently introduce malware or facilitate unauthorized access. Managing supply chain risks requires thorough vetting of vendors, including assessing their cybersecurity practices and compliance with regulations. Contracts should include provisions for data protection, breach notification, and liability. Additionally, healthcare organizations should regularly monitor and audit vendor activities to ensure ongoing compliance and security.^[15,16]^ To address these diverse cybersecurity threats, healthcare organizations must adopt a multi-layered approach to security. This approach includes technical measures, such as firewalls, intrusion detection systems, and encryption, as well as administrative measures, such as policies, training, and incident response planning. Regular risk assessments are essential for identifying vulnerabilities and implementing appropriate controls. Given the dynamic nature of cyber threats, healthcare organizations must also stay informed about emerging risks and continuously update their security measures. Collaboration with external experts, such as cybersecurity firms and regulatory bodies, can provide valuable insights and support in strengthening security posture.^[17,18]^

## 5. Regulatory framework and regulations

At the international level, the GDPR of the European Union (EU) stands as a comprehensive legal framework aimed at protecting personal data and privacy. The GDPR imposes strict requirements on data controllers and processors, including healthcare providers, concerning collecting, processing, and storing personal data.^[19]^ It mandates that organizations implement appropriate technical and organizational measures to secure personal data, conduct regular risk assessments, and ensure data confidentiality, integrity, and availability. Notably, the GDPR requires that data breaches be reported to relevant authorities within 72 hours of discovery, which places a significant compliance burden on healthcare entities.^[20,21]^ The regulation also grants patients the right to access their data, request corrections, and, in certain cases, delete their data, reinforcing patient autonomy and control over personal information. The primary regulatory Framework for healthcare data protection in the United States is the HIPAA. Enacted in 1996, HIPAA sets national standards for protecting health information, mandating the safeguarding of electronic protected health information (ePHI) through the HIPAA Security Rule.^[22,23]^ The Security Rule requires healthcare providers and their business associates to implement administrative, physical, and technical safeguards to protect ePHI. This includes access controls, encryption, audit controls, and transmission security. HIPAA also includes a Breach Notification Rule, which obligates covered entities to notify affected individuals, the Department of Health and Human Services (HHS), and, in some cases, the media in the event of a data breach.^[24]^ Penalties for noncompliance with HIPAA can be severe, including substantial fines and potential criminal charges, highlighting the importance of adherence to these regulations for healthcare providers. Beyond the GDPR and HIPAA, many other countries have established data protection laws impacting healthcare cybersecurity. For example, Canada’s Personal Information Protection and Electronic Documents Act (PIPEDA) regulates the private sector’s collection, use, and disclosure of personal information, including healthcare.^[25]^ PIPEDA mandates that organizations obtain informed consent for data collection, implement security safeguards, and provide individuals access to their personal information. Like the GDPR, PIPEDA requires organizations to notify individuals of any breaches of security safeguards that pose a real risk of significant harm.^[26]^ In Australia, the Privacy Act 1988 and the subsequent Notifiable Data Breaches (NDB) scheme provide the legal Framework for data protection. The NDB scheme requires organizations, including healthcare providers, to notify individuals and the Office of the Australian Information Commissioner (OAIC) when a data breach is likely to result in serious harm.^[27]^ The scheme emphasizes the importance of timely breach notification and transparency in handling personal information. Asian countries have also been active in developing data protection regulations. Japan’s Act on the Protection of Personal Information (APPI) and South Korea’s Personal Information Protection Act are notable examples. These laws set stringent requirements for handling personal information, including provisions for data security, breach notification, and the rights of data subjects.^[28,29]^ In Japan, the Personal Information Protection Commission (PIPC) oversees compliance with the APPI, while in South Korea, the PIPC performs a similar role under the Personal Information Protection Act. Emerging technologies, such as AI and quantum computing, present new challenges and considerations for regulatory frameworks.^[30]^ AI systems, which can analyze vast amounts of healthcare data to assist in diagnosis and treatment, raise concerns about data security, privacy, and ethical implications. The potential for AI to introduce biases or make decisions that lack transparency necessitates robust regulatory oversight. The European Union has been proactive in this area, proposing the AI Act, which aims to regulate the use of AI, including in healthcare, by categorizing AI systems based on their risk levels and setting compliance requirements accordingly.^[31]^ The AI Act seeks to ensure that high-risk AI systems, such as those used in healthcare, meet strict data protection and transparency standards. Quantum computing, with its potential to revolutionize data encryption and decryption, poses significant challenges to cybersecurity frameworks. Current encryption methods, which rely on the computational difficulty of certain mathematical problems, may become obsolete with the advent of quantum computers capable of solving these problems rapidly.^[32]^ This necessitates the development of new cryptographic methods, often called post-quantum cryptography, to protect sensitive data.^[33]^ Regulatory bodies are beginning to consider the implications of quantum computing for data security. They encourage research and development to ensure that future technologies can safeguard against quantum threats. The regulatory landscape is further complicated by the global nature of cybersecurity threats and the varying legal requirements across jurisdictions. International cooperation and harmonization of regulations are essential to address cross-border data flows and ensure comprehensive protection of personal information.^[34]^ Organizations such as the International Organization for Standardization (ISO) and the International Electrotechnical Commission have developed international standards, such as ISO/IEC 27001, which provide a framework for information security management systems.^[12]^ These standards help organizations implement best cybersecurity practices and comply with international expectations. For clinicians and healthcare organizations, navigating these complex regulatory frameworks requires a thorough understanding of the specific requirements in their jurisdiction and an awareness of international standards. This includes staying informed about updates to laws and regulations, implementing appropriate security measures, and ensuring that all staff are trained in data protection practices. Legal compliance is a matter of adhering to statutory obligations and is critical to maintaining patient trust and safeguarding sensitive health information.^[35–37]^

## 6. Comparative analysis of national regulations on cybersecurity in healthcare

In the United States, healthcare cybersecurity is primarily governed by the HIPAA and its associated Privacy and Security Rules. HIPAA mandates strict standards for protecting patient health information, including EHRs.^[38]^ The Security Rule requires healthcare organizations to implement administrative, physical, and technical safeguards to protect ePHI. Clinicians are legally obligated to adhere to these standards, and failure to comply can result in significant penalties, including fines and potential criminal charges. The Office for Civil Rights within the Department of HHS oversees the enforcement of HIPAA regulations.^[39]^ Additionally, the Health Information Technology for Economic and Clinical Health (HITECH) Act further strengthens data protection by promoting meaningful use of EHRs and introducing breach notification requirements.^[1,2]^ In the European Union, the GDPR is the primary legislation governing cybersecurity in healthcare. GDPR applies to all organizations processing personal data of EU residents, including healthcare providers. It imposes stringent requirements on data controllers and processors, mandating data protection by design and default and specific health data provisions categorized as sensitive.^[40,41]^ Healthcare organizations must implement comprehensive data protection measures and ensure patients have control over their data. GDPR also introduces significant penalties for noncompliance, with fines reaching up to 4% of annual global turnover. For clinicians, adherence to GDPR means rigorous data handling procedures and a clear understanding of patients’ rights concerning their data.^[3,4]^ The United Kingdom, having implemented GDPR into national law as the UK General Data Protection Regulation (UK GDPR) alongside the Data Protection Act 2018, follows a similar approach to data protection as the EU. The UK GDPR governs the processing of personal data, including health information, focusing on transparency, accountability, and patient consent.^[42]^ The Information Commissioner’s Office enforces data protection laws and can impose fines for breaches. Clinicians in the UK are required to comply with these regulations, ensuring that patient data is processed lawfully and securely. The National Health Service (NHS) also provides additional guidance on cybersecurity practices and data protection standards.^[5,6]^ Canada’s approach to healthcare cybersecurity is governed by the Personal Information Protection and Electronic Documents Act (PIPEDA) and the provincial Personal Health Information Protection Acts (PHIPAs). PIPEDA sets out principles for collecting, using, and disclosing personal information in commercial activities, including healthcare. Provincial legislation, such as Ontario’s PHIPA, provides more specific guidelines for health information.^[43]^ These laws require healthcare organizations to implement security measures to protect patient data and to notify individuals of breaches. Clinicians must adhere to federal and provincial regulations, ensuring that patient information is protected and breaches are reported per legal requirements.^[7,8]^ In Australia, healthcare cybersecurity is regulated by the Privacy Act 1988 and the Australian Privacy Principles (APPs). The Privacy Act outlines the obligations of healthcare providers regarding handling personal information, including health data. The APPs require healthcare organizations to adopt reasonable steps to protect personal data from unauthorized access or disclosure. Additionally, the NDB scheme mandates that organizations report certain data breaches to affected individuals and the OAIC. Clinicians in Australia must follow these regulations to ensure compliance with data protection and breach notification requirements.^[9,10]^ Japan’s healthcare cybersecurity regulations are guided by the APPI and related guidelines. The APPI establishes rules for handling personal information, including health data, and requires organizations to implement measures to ensure data security. The PIPC oversees compliance and enforcement. Clinicians in Japan must comply with these regulations, implementing appropriate security measures to protect patient data and responding to data breaches per the law.^[11,12]^ China has introduced comprehensive data protection regulations through the Cybersecurity and Personal Information Protection Law (PIPL). The Cybersecurity Law focuses on protecting critical information infrastructure, including healthcare systems, and mandates data security measures for operators. The PIPL, which came into effect in 2021, regulates the processing of personal information and provides detailed requirements for data protection. Various regulatory bodies, including the Cyberspace Administration of China, enforce these laws. Clinicians in China must adhere to these stringent regulations, ensuring robust cybersecurity practices and compliance with data protection standards.^[13,14]^ In India, healthcare cybersecurity is governed by the Information Technology Act 2000 (IT Act) and the proposed Personal Data Protection Bill (PDPB). The IT Act includes provisions for cybersecurity and the protection of electronic data. At the same time, the PDPB, which is expected to be enacted soon, will introduce comprehensive data protection requirements, including those specific to health data. Healthcare organizations and clinicians in India must comply with these regulations, implement security measures to protect patient data, and respond to breaches by the new legal Framework.^[15,16]^ Table 1 compares the analysis of the primary legislation, data protection focus, enforcement bodies, penalties for noncompliance, and key legal implications for clinicians in 8 jurisdictions. This table highlights the distinct and overlapping aspects of cybersecurity regulations in healthcare across the United States, European Union, United Kingdom, Canada, Australia, Japan, China, and India. It emphasizes the varying approaches to protecting personal and health information, the associated enforcement mechanisms, and clinician responsibilities. The diverse regulatory frameworks underline healthcare professionals need to be well-versed in local laws to ensure compliance and effectively protect patient data.

**Table 1:** Comparative analysis of healthcare cybersecurity regulations and legal implications for clinicians across various jurisdictions.

Country	Primary legislation	Data protection focus	Enforcement body	Penalties for noncompliance	Key legal implications for clinicians
United States	HIPAA, HITECH Act	PHI protection, breach notifications	Office for Civil Rights (OCR)	Fines, criminal charges	Strict adherence to safeguards, breach reporting requirements
European Union	GDPR	Personal data, sensitive health data	Data Protection Authorities (DPAs)	Fines up to 4% of global turnover	Comprehensive data protection, patient consent, breach reporting
United Kingdom	UK GDPR, Data Protection Act 2018	Personal data, health information	Information Commissioner’s Office (ICO)	Fines, enforcement actions	Compliance with data protection and patient consent requirements
Canada	PIPEDA, PHIPAs (provincial)	Personal and health information	Office of the Privacy Commissioner (OPC)	Fines, legal actions	Adherence to federal and provincial regulations, breach notifications
Australia	Privacy Act 1988, APPs, NDB scheme	Personal information, health data	Office of the Australian Information Commissioner (OAIC)	Fines, enforcement actions	Compliance with privacy principles, breach reporting
Japan	APPI	Personal and health information	Personal Information Protection Commission (PIPC)	Fines, compliance orders	Implementation of data security measures, breach response
China	Cybersecurity Law, PIPL	Critical information infrastructure, personal data	Cyberspace Administration of China (CAC)	Fines, operational restrictions	Adherence to stringent data protection and security measures
India	IT Act, proposed PDPB	Electronic data, personal and health information	Ministry of Electronics and IT	Fines, legal actions	Compliance with cybersecurity requirements, breach management

The healthcare cybersecurity approach varies significantly across countries, reflecting different regulatory philosophies and legal frameworks. While there are commonalities, such as the emphasis on protecting personal and health information, the specific requirements and enforcement mechanisms differ. Clinicians globally must navigate these diverse regulations, ensuring compliance with local laws and implementing effective cybersecurity measures to safeguard patient data and maintain the integrity of healthcare services. The evolving nature of cyber threats necessitates continuous adaptation, vigilance, and collaboration between healthcare providers, regulators, and cybersecurity experts to address the challenges of protecting sensitive health information in the digital age.

APPs = Australian Privacy Principles, HIPAA = Health Insurance Portability and Accountability Act, HITECH = Health Information Technology for Economic and Clinical Health, PDPB = Personal Data Protection Bill, PHI = protected health information.

## 7. Pioneering work

The field of healthcare cybersecurity has seen numerous pioneering contributions that have shaped its current landscape. One of the earliest and most significant contributions to cybersecurity in healthcare was the development of encryption techniques for securing medical data.^[40]^ Encryption has been a cornerstone of data protection, providing a mechanism to safeguard information from unauthorized access. The work of Whitfield Diffie and Martin Hellman in the mid-1970s introduced the concept of public-key cryptography, which revolutionized the field by enabling secure communication over unsecured channels.^[41]^ This foundational work laid the groundwork for secure data transmission, critical in healthcare settings where patient confidentiality is paramount. Building on this, the advent of the Advanced Encryption Standard in the early 2000s provided a robust and efficient algorithm for encrypting EHRs, ensuring data integrity and confidentiality.^[42]^ The introduction of the HIPAA in the United States marked a significant regulatory milestone in healthcare cybersecurity. Enacted in 1996, HIPAA established national standards for protecting health information, mandating the implementation of administrative, physical, and technical safeguards.^[43]^ The HIPAA Security Rule, a component of this legislation, specifically addressed the need for healthcare providers to secure ePHI. This regulation emphasized the importance of encryption and access controls. It introduced the concept of risk assessments, which has since become a standard practice in identifying and mitigating cybersecurity threats in healthcare.^[44]^ HIPAA’s influence extended beyond the United States, serving as a model for data protection laws in other jurisdictions. Another pioneering work in healthcare cybersecurity was the development of intrusion detection systems (IDS) tailored for medical environments. As healthcare organizations increasingly adopted digital systems, the need for specialized IDS to detect and respond to unauthorized access or malicious activity became apparent. The work of Dorothy E. Denning in the 1980s on anomaly detection laid the theoretical foundation for IDS.^[45]^ In healthcare, these systems are crucial for monitoring network traffic, detecting unusual patterns, and alerting administrators to potential breaches. This proactive approach to security is essential for protecting sensitive patient data and ensuring the continuous operation of healthcare services. The rise of telemedicine and remote patient monitoring has further underscored the importance of secure communication channels in healthcare. The pioneering work of researchers in developing Virtual Private Networks and secure socket layer protocols has been critical in this regard. Virtual Private Networks provide a secure tunnel for transmitting patient data over the Internet, while secure socket layer ensures that communications between healthcare providers and patients are encrypted and authenticated.^[46]^ These technologies have enabled the safe exchange of medical information, facilitating remote consultations and improving access to healthcare services. In recent years, AI has emerged as a transformative technology in healthcare cybersecurity. Pioneering work in AI-based threat detection systems has significantly enhanced the ability to identify and respond to cyber threats. Machine learning algorithms can analyze vast amounts of data to detect anomalies and predict potential security incidents. The work of researchers like Ian Goodfellow on generative adversarial networks has been particularly influential in advancing AI’s capabilities in cybersecurity.^[47]^ In healthcare, AI-powered systems can identify unusual patterns in network traffic or user behavior, flagging potential security breaches before they occur. Moreover, AI can assist in automating incident response, reducing the time needed to contain and mitigate cyber threats. Blockchain technology represents another pioneering area in healthcare cybersecurity. Initially popularized as the underlying technology for cryptocurrencies, blockchain has found applications in securing medical records and ensuring data integrity. Satoshi Nakamoto work in creating Bitcoin introduced the concept of a decentralized ledger, where transactions are recorded in a tamper-proof manner.^[48]^ In healthcare, blockchain can create immutable patient data records, ensuring that information is accurate and unaltered. This is particularly valuable in managing medical records across different institutions and ensuring patients have control over their data. Additionally, blockchain can facilitate the secure and transparent sharing of health information, essential for research and collaborative care. The European Union’s GDPR has been another pioneering regulatory framework with significant implications for healthcare cybersecurity. Implemented in 2018, the GDPR set a new data protection and privacy standard, imposing stringent requirements on organizations that handle personal data.^[49]^ The regulation’s focus on user consent, data minimization, and the right to be forgotten has profoundly impacted how healthcare organizations manage patient data. The GDPR’s influence extends globally, as organizations outside the EU that deal with EU citizens’ data must also comply with its provisions. This regulation has heightened awareness of data protection issues and driven the adoption of more robust cybersecurity measures in healthcare. Emerging technologies such as quantum computing pose opportunities and challenges for healthcare cybersecurity. Pioneering work in quantum cryptography, such as the development of quantum key distribution (QKD), offers potential solutions for securing data against future threats posed by quantum computers.^[50]^ QKD enables the creation of secure communication channels theoretically immune to eavesdropping, making it a promising technology for protecting sensitive medical data. However, the development of quantum computers also threatens to break current encryption methods, necessitating further research and innovation in post-quantum cryptography. Implementing multi-factor authentication (MFA) systems has also been a key advancement in healthcare cybersecurity. Pioneering efforts in this area have focused on developing authentication mechanisms beyond traditional username and password systems. MFA typically combines something the user knows (like a password), something the user has (like a smartphone or token), and something the user is (biometrics).^[51]^ This layered approach significantly enhances security by making it more difficult for unauthorized users to access sensitive systems. MFA protects access to electronic health records and other critical systems. The role of healthcare organizations and governmental bodies in advancing cybersecurity cannot be understated. Initiatives such as the Health Information Trust Alliance (HITRUST) and the National Institute of Standards and Technology (NIST) Cybersecurity Framework have provided valuable guidance and standards for healthcare organizations.^[52,53]^ HITRUST, for example, has developed a comprehensive framework that integrates various cybersecurity standards and regulations, helping healthcare providers assess and improve their cybersecurity posture. With its core functions of Identifying, Protecting, Detecting, Responding, and Recovering, the NIST Cybersecurity Framework provides a structured approach to managing cybersecurity risks, applicable across various sectors, including healthcare.

## 8. Novel insights

A significant novel insight in healthcare cybersecurity is the growing recognition of the importance of a holistic approach to security. Rather than focusing solely on technical solutions, there is an increasing emphasis on integrating technical, organizational, and human factors to create a comprehensive security framework.^[54]^ This holistic approach acknowledges that encryption and firewalls are crucial but must be complemented by strong governance structures, clear policies, and an informed and vigilant workforce. This perspective is particularly relevant given the high number of cybersecurity incidents attributed to human error, such as phishing attacks and insider threats. Training and awareness programs for healthcare staff are now vital components of cybersecurity strategies, ensuring that individuals at all levels understand their role in protecting patient data.^[51]^ The advent of AI and ML has brought novel insights into threat detection and response. AI-powered systems have demonstrated the ability to analyze large volumes of data and detect patterns indicative of cyber threats more accurately and swiftly than traditional methods. For instance, deep learning algorithms can identify subtle anomalies in network traffic or user behavior that might indicate a potential security breach. The application of AI in cybersecurity also extends to predictive analytics, where ML models predict future threats based on historical data. This proactive approach enables healthcare organizations to anticipate and mitigate risks before they manifest, significantly reducing the likelihood of successful cyber attacks.^[52]^ However, integrating AI into healthcare cybersecurity raises algorithmic bias and transparency concerns, necessitating further research into ethical AI practices. Blockchain technology represents another novel area of exploration in healthcare cybersecurity. Originally developed as a decentralized ledger for cryptocurrencies, blockchain’s inherent properties of immutability and transparency offer promising applications in securing healthcare data. One of the most significant insights is the potential of blockchain to provide a secure and auditable trail of transactions, ensuring the integrity of patient records and medical histories. By decentralizing data storage, blockchain reduces the risk of centralized attacks and unauthorized alterations. Additionally, smart contracts—self-executing contracts with the terms of the agreement directly written into code—can automate and enforce compliance with data protection regulations, streamlining processes such as consent management and data sharing among healthcare providers.^[53]^ Another critical area of novel insight is the development of advanced encryption techniques tailored to the unique needs of healthcare data. While traditional encryption methods like Advanced Encryption Standard and RSA remain foundational, new approaches such as homomorphic encryption and quantum-resistant cryptography are gaining attention. Homomorphic encryption allows computations on encrypted data without decrypting it, thereby preserving privacy while enabling analysis. This capability is particularly valuable in healthcare for performing secure data analytics and researching sensitive patient information. Quantum-resistant cryptography addresses the looming threat of quantum computing, which could potentially break current encryption methods. Researchers are exploring new cryptographic algorithms that can withstand quantum attacks, ensuring the long-term security of healthcare data.^[54]^ The novel insights into cybersecurity are not limited to technical advancements; regulatory frameworks and legal considerations have also evolved significantly. The GDPR in the European Union and the HIPAA in the United States have set high standards for data protection in healthcare. A novel aspect of these regulations is their extraterritorial application, meaning they can affect entities outside their jurisdictions if they handle the data of EU or US citizens. This global reach underscores the importance of a comprehensive understanding of international data protection laws for healthcare organizations. Furthermore, the concept of data sovereignty—where data is subject to the laws of the country in which it is stored—has gained prominence. This has increased the emphasis on data localization and the need for healthcare organizations to navigate complex legal landscapes to ensure compliance.^[55]^ Another area of emerging insight is the role of cyber insurance in healthcare. As cyber threats become more sophisticated and pervasive, healthcare organizations increasingly turn to cyber insurance as a risk management tool. Cyber insurance policies can cover various incidents, including data breaches, ransomware attacks, and business interruption. The novel aspect of cyber insurance lies in its potential to influence cybersecurity practices positively. Insurers often require organizations to meet specific cybersecurity standards as a condition of coverage, thereby incentivizing the implementation of robust security measures. Additionally, the claims process following a cybersecurity incident can provide valuable insights into vulnerabilities and areas for improvement, contributing to a culture of continuous improvement in cybersecurity practices.^[56]^ The exploration of patient-centric approaches in cybersecurity has also yielded novel insights. Traditionally, cybersecurity efforts have focused on protecting healthcare systems and providers. However, there is a growing recognition of the importance of empowering patients to protect their data. This includes giving patients greater control over their health information and enabling them to access, correct, and manage their data securely. Personal health records (PHRs) and mobile health applications are examples of tools that can facilitate this empowerment. However, these tools also introduce new security challenges, as they may be vulnerable to cyber-attacks. Ensuring the security of patient-facing technologies and educating patients about best practices for protecting their data are crucial components of a comprehensive cybersecurity strategy.^[57]^ The intersection of cybersecurity and patient safety has also emerged as a critical area of focus. Cybersecurity incidents can directly affect patient safety, particularly in cases where medical devices or critical systems are compromised. For example, ransomware attacks that disrupt access to electronic health records or diagnostic equipment can delay treatment and jeopardize patient outcomes. Recognizing this, a growing emphasis is on integrating cybersecurity into patient safety frameworks and protocols. This includes conducting regular risk assessments, developing incident response plans, prioritizing patient safety, and fostering collaboration between IT and clinical teams. By aligning cybersecurity with patient safety objectives, healthcare organizations can ensure a more resilient and secure environment for patient care.^[58]^ Finally, the novel insights into healthcare cybersecurity have also led to developing new frameworks and models for evaluating and managing cybersecurity risks. One such model is the zero trust architecture, which operates on the principle of “never trust, always verify.” In a zero-trust model, all users and devices are treated as potential threats, regardless of their location within or outside the network. This approach emphasizes strict access controls, continuous monitoring, and micro-segmentation to limit the lateral movement of threats within a network. Adopting zero trust architecture in healthcare is particularly relevant given the increasing use of remote and mobile devices, which can introduce new vulnerabilities. By implementing zero trust principles, healthcare organizations can enhance their security posture and reduce the risk of data breaches.^[59]^

## 9. Critical analysis

One of the primary concerns in healthcare cybersecurity is the protection of sensitive patient data. The healthcare sector is a prime target for cybercriminals due to the valuable nature of medical data, which includes personal, financial, and health-related information. Despite implementing various security measures, data breaches remain prevalent, highlighting the limitations of current approaches.^[60]^ For example, traditional methods such as firewalls, antivirus software, and simple encryption are increasingly insufficient against sophisticated threats like advanced persistent threats and zero-day exploits. These sophisticated attacks often bypass conventional defenses, exploiting software and network architecture vulnerabilities. The healthcare industry’s reliance on legacy systems, which may not support modern security protocols, exacerbates this issue. As a result, there is a critical need for continuous monitoring and updating of security measures to address evolving threats.^[1,2]^ The role of human factors in cybersecurity cannot be overstated. Many cybersecurity incidents in healthcare result from human error, such as phishing attacks, weak password practices, and mishandling of sensitive information. These incidents underscore the importance of comprehensive cybersecurity training and awareness programs for healthcare professionals. However, implementing such programs faces several challenges, including the diversity of staff roles, varying levels of technical expertise, and the demanding nature of healthcare work that often leaves little time for additional training. While some organizations have successfully integrated cybersecurity training into their routine operations, there is no standardized approach, leading to inconsistent levels of preparedness across the industry. Moreover, the effectiveness of these programs in changing behaviors and reducing risks remains difficult to quantify, necessitating further research into best practices for cybersecurity education.^[3]^ Another critical aspect of healthcare cybersecurity is the regulatory environment. Regulations such as the HIPAA in the United States and the GDPR in the European Union provide frameworks for protecting patient data. However, these regulations also present challenges. For instance, the strict requirements of GDPR, including the right to be forgotten and the obligation to report data breaches within 72 hours, can be difficult for healthcare organizations, especially smaller entities with limited resources. Additionally, the global nature of healthcare data—where patient information often crosses borders—complicates compliance with multiple, sometimes conflicting, regulatory regimes. This situation raises questions about the adequacy of current legal frameworks in addressing the global nature of cyber threats. While international cooperation and harmonization of regulations are often suggested as solutions, achieving consensus on standards and enforcement mechanisms is complex and politically challenging.^[4,5]^ The ethical considerations in healthcare cybersecurity are equally critical. The principle of patient autonomy, which emphasizes patients’ right to control their health information, is frequently challenged in the digital age. For example, using big data analytics and AI in healthcare often involves aggregating and analyzing large datasets, potentially including identifiable patient information. While these technologies can lead to valuable insights and improvements in patient care, they also raise concerns about privacy, consent, and the potential for misuse of data. The ethical implications are further complicated when considering AI’s role in decision-making, where algorithm biases can lead to unequal treatment or misdiagnosis. Ensuring transparency, accountability, and fairness in using AI and other digital tools in healthcare is essential for maintaining public trust and adhering to ethical standards.^[6]^ Emerging technologies, such as blockchain and quantum computing, offer promising solutions to some challenges in healthcare cybersecurity. With its decentralized and immutable ledger, blockchain can enhance data security and integrity, making it harder for cybercriminals to alter or access sensitive information. However, the adoption of blockchain in healthcare is still in its infancy, with several technical and regulatory hurdles to overcome. For instance, blockchain systems’ scalability and integration with existing healthcare infrastructures are ongoing challenges. Quantum computing, while still largely theoretical in its application, poses both opportunities and threats. On the one hand, quantum-resistant cryptography is being developed to protect against future quantum attacks that could break current encryption methods. On the other hand, the potential for quantum computing to disrupt existing security frameworks necessitates a proactive approach to developing new cryptographic techniques and standards.^[7,8]^ The increasing adoption of the Internet of Things (IoT) in healthcare, including wearable devices and connected medical equipment, further complicates cybersecurity. While IoT devices offer significant benefits regarding patient monitoring and personalized care, they also introduce new vulnerabilities. Many IoT devices lack robust security features, making them easy cyberattack targets. The proliferation of these devices creates a vast and expanding attack surface that healthcare organizations must manage. Moreover, the interconnectivity of IoT devices means that a breach in one device can potentially compromise the entire network, posing risks not only to data security but also to patient safety. Addressing these challenges requires a comprehensive approach, including the development of industry-wide standards for IoT security, regular device monitoring, and updates, and clear guidelines for manufacturers and healthcare providers.^[9]^ Cyber insurance has emerged as a critical component of risk management in healthcare cybersecurity. Insurance policies can help organizations mitigate the financial impact of cyber incidents, covering costs related to data breaches, ransomware attacks, and other cyber threats. However, the cyber insurance market is still developing, with insurers facing challenges in accurately assessing risks and determining premiums. Moreover, cyber insurance may inadvertently encourage complacency among healthcare organizations, who might rely on insurance rather than investing in robust cybersecurity measures. Insurers, therefore, play a crucial role in setting cybersecurity standards by requiring policyholders to implement specific security practices as a condition of coverage. This dynamic creates a feedback loop where the evolving nature of cyber threats influences insurance policies and vice versa, shaping the overall cybersecurity landscape in healthcare.^[10]^ The relationship between cybersecurity and patient safety is another critical area of analysis. Cybersecurity incidents can directly and severely impact patient safety, especially involving medical devices or critical healthcare infrastructure. For example, ransomware attacks that lock healthcare providers out of their systems can delay diagnosis and treatment, potentially leading to adverse patient outcomes. Similarly, cyberattacks targeting medical devices, such as pacemakers or insulin pumps, can threaten patients’ lives. Recognizing the intersection of cybersecurity and patient safety, healthcare organizations must prioritize both as interconnected elements of their overall risk management strategy. This includes conducting regular risk assessments, developing incident response plans prioritizing patient safety, and fostering collaboration between cybersecurity and clinical teams.^[11]^

## 10. Engagement with counterarguments

In healthcare cybersecurity discourse, various counterarguments arise regarding the necessity, feasibility, and implementation of robust security measures. While the prevailing narrative emphasizes the critical importance of protecting patient data and maintaining system integrity, alternative viewpoints suggest that the focus on cybersecurity may be exaggerated, excessively costly, or technically infeasible in certain contexts.^[61]^ One common counterargument is that the emphasis on cybersecurity in healthcare might overshadow the primary goal of medical care, which is to provide timely and effective treatment to patients. Critics argue that the extensive focus on securing digital systems and data can lead to overemphasizing technical aspects at the expense of clinical care. They contend that financial and human resources could be better allocated to improving medical services rather than investing heavily in cybersecurity infrastructure. However, this perspective overlooks the interconnectedness of cybersecurity and patient safety. Cybersecurity incidents, such as ransomware attacks or breaches of medical device security, can directly impact patient care by delaying treatments, disrupting medical procedures, or compromising the accuracy of medical records. Therefore, adequate cybersecurity measures are not a diversion from patient care but a fundamental aspect of ensuring safe and effective medical services.^[1,2]^ Another argument is that the costs associated with implementing comprehensive cybersecurity measures in healthcare are prohibitive, especially for smaller institutions with limited budgets. It is argued that the financial burden of acquiring advanced cybersecurity tools, hiring specialized personnel, and conducting regular security assessments can be overwhelming for smaller hospitals and clinics. This viewpoint is not without merit, as the healthcare industry does face significant financial constraints, and the cost of cybersecurity can be substantial. However, the cost of a cybersecurity incident can far exceed the expenses associated with preventive measures. Data breaches, for example, can lead to legal penalties, loss of patient trust, and substantial recovery costs. Moreover, government incentives, grants, and cybersecurity frameworks are increasingly available to support healthcare organizations in enhancing their security posture. Thus, while the financial challenge is real, the long-term benefits and potential cost savings of investing in cybersecurity outweigh the initial expenditures.^[3,4]^ A third counterargument posits that healthcare organizations, especially smaller ones, may lack the technical expertise and resources to implement sophisticated cybersecurity measures. The rapidly evolving nature of cyber threats and the complexity of securing digital systems can be daunting, leading to a perception that achieving adequate cybersecurity is an insurmountable challenge. This perspective is often grounded in the reality that many healthcare organizations still rely on outdated technology and lack specialized IT staff. However, this argument fails to account for the diverse range of cybersecurity solutions available today, catering to varying technical capability and budget levels. For instance, cloud-based security services, managed security providers, and standardized cybersecurity protocols can offer scalable solutions that are accessible even to smaller healthcare providers. Moreover, collaborative initiatives and partnerships with larger healthcare systems or cybersecurity firms can provide smaller institutions with the necessary expertise and resources.^[5,6]^ Some stakeholders argue that the regulatory landscape surrounding healthcare cybersecurity is overly complex and burdensome. They claim that the diversity of regulations, such as HIPAA in the United States and GDPR in Europe, creates a confusing and often contradictory compliance environment. This argument suggests that the stringent requirements imposed by these regulations can stifle innovation and impose unnecessary administrative burdens on healthcare providers. While there is validity to the concern about regulatory complexity, it is important to recognize that these regulations are designed to protect patient privacy and data security. Rather than viewing regulations as impediments, they should be seen as frameworks that set minimum standards for data protection. Moreover, regulatory bodies and professional associations increasingly offer guidance and support to help healthcare organizations navigate compliance requirements. Simplification and harmonization of regulations are ongoing discussions that can alleviate perceived burdens while maintaining robust data protection standards.^[7,8]^ Another critical counterargument revolves around the balance between data accessibility and security. In healthcare, timely access to patient data is crucial for effective diagnosis and treatment. Critics argue that stringent cybersecurity measures, such as multi-factor authentication and data encryption, can sometimes hinder rapid patient information access, particularly in emergencies. They suggest that imposing rigorous security protocols could delay critical care, potentially compromising patient outcomes. However, this argument often stems from a misunderstanding of modern cybersecurity practices, which aim to balance security with usability. Technologies such as single sign-on (SSO) systems and secure mobile access have been developed to streamline access while maintaining security. Moreover, the healthcare sector can adopt role-based access controls and audit trails to ensure efficient and secure access to sensitive information. The goal is not to create barriers but to implement measures that protect patient data without impeding healthcare delivery.^[9,10]^ The issue of ethical considerations in cybersecurity is another area where counterarguments arise. Some argue that the extensive monitoring and surveillance required for effective cybersecurity can infringe on patients’ and healthcare providers’ privacy and autonomy. For instance, using monitoring software to detect unusual access patterns or potential insider threats can be seen as invasive. This concern raises important ethical questions about how surveillance is justified in the name of security. However, balancing these concerns with the need to protect sensitive health information is essential. Ethical frameworks and oversight mechanisms can help ensure that cybersecurity practices are implemented in a way that respects individual rights and privacy. Transparency about the types and purposes of monitoring and ensuring data collection is proportional to the security risk can help mitigate ethical concerns.^[11,12]^ The role of emerging technologies, such as AI and ML, in healthcare cybersecurity also sparks debate. Some critics argue that these promising technologies are not yet mature enough to be relied upon for critical cybersecurity functions. They point to issues such as the potential for AI algorithms to produce false positives, the risk of AI systems being manipulated by adversarial attacks, and the lack of transparency in AI decision-making processes. These concerns are valid, as the deployment of AI and ML in cybersecurity is still in its developmental stages. However, dismissing these technologies outright overlooks their potential benefits. AI and ML can enhance threat detection capabilities, automate routine security tasks, and provide predictive analytics to identify potential vulnerabilities. The key is to integrate these technologies thoughtfully, with a clear understanding of their limitations and an emphasis on human oversight and control.^[13,14]^ Finally, there is a philosophical counterargument regarding the inevitability of cyberattacks. Some stakeholders argue that, regardless of the measures taken, healthcare systems will always be vulnerable to some degree of cyber risk. This perspective, often termed “cyber fatalism,” suggests that the focus should not solely be on prevention but also resilience and recovery. While it is true that no system can be entirely immune to cyber threats, this viewpoint risks fostering complacency. The focus should indeed encompass resilience—developing robust incident response plans, ensuring data backups, and maintaining business continuity—but it should not downplay the importance of proactive prevention measures. A comprehensive cybersecurity strategy should integrate preventive and reactive elements, continually adapting to the evolving threat landscape.^[15,16]^

## 11. Clinicians’ duty of care

The obligation to provide competent and evidence-based medical treatment is at the core of the clinician’s duty of care. This duty necessitates that clinicians stay abreast of current medical knowledge and advancements, applying the best available evidence to patient care. The dynamic nature of medical science means that standards of care are continually evolving, requiring clinicians to engage in lifelong learning and professional development. For instance, introducing new diagnostic technologies, treatments, and guidelines necessitates clinicians regularly updating their knowledge and skills. Failure to do so can lead to outdated or suboptimal care, potentially harming patients. Moreover, clinicians must exercise sound clinical judgment, tailoring treatments to patients’ needs, which requires a deep understanding of general medical knowledge and specific patient contexts.^[1,2]^ Another critical component of the duty of care is maintaining patient confidentiality. The clinician-patient relationship is fundamentally built on trust, with patients expecting their personal and medical information handled with the utmost discretion. Confidentiality is an ethical obligation and a legal requirement in many jurisdictions, governed by laws such as the HIPAA in the United States and the GDPR in Europe. Breaching confidentiality can lead to severe consequences, including legal penalties, professional licensure loss, and patient trust erosion. However, confidentiality is not absolute; exceptions may arise, such as when there is a duty to warn others about potential harm or when disclosure is required by law. Navigating these exceptions requires careful ethical consideration and a balanced approach that respects patient privacy while fulfilling legal and professional obligations.^[3,4]^ Informed consent is another fundamental aspect of the clinician’s duty of care. It involves providing patients with adequate information about their diagnosis, treatment options, risks, benefits, and alternatives, allowing them to make informed decisions about their care. Informed consent is rooted in the ethical principles of autonomy and respect for persons, recognizing the right of individuals to make decisions about their bodies and health. Clinicians must ensure patients comprehend the information provided, which may involve addressing language barriers, literacy levels, and cultural differences. Obtaining informed consent is not merely a procedural formality but a dialogue that fosters mutual understanding and respects the patient’s values and preferences. It also ensures that consent is voluntarily given, free from coercion or undue influence. In cases where patients cannot provide informed consent, such as in emergencies or when dealing with minors or incapacitated individuals, clinicians must seek consent from legally authorized representatives or act in the patient’s best interests based on clinical judgment and ethical considerations.^[5,6]^ The duty of care also extends to clinicians’ professional behavior and ethical conduct. This includes maintaining a high standard of professionalism and demonstrating compassion, integrity, and respect in all interactions with patients and colleagues. Professionalism involves adhering to ethical codes and guidelines by medical organizations and regulatory bodies. For example, clinicians are expected to avoid conflicts of interest, refrain from exploiting patients for personal gain, and report any unethical behavior observed in the workplace. Ethical dilemmas frequently arise in clinical practice, such as decisions around end-of-life care, resource allocation, and patient autonomy versus beneficence. Clinicians must navigate these dilemmas thoughtfully, often consulting ethical frameworks, professional guidelines, and, where appropriate, ethics committees. Making ethical decisions and acting with moral courage is critical to the clinician’s duty of care.^[7,8]^ Continuity of care is another essential aspect of the clinician’s duty. This concept involves providing seamless and coordinated care throughout the patient’s healthcare journey, ensuring that transitions between different providers or healthcare settings do not compromise the quality of care. To facilitate continuity, clinicians must communicate effectively with other healthcare professionals, including specialists, nurses, and allied health practitioners. This communication is particularly crucial when patients are transferred between settings, such as from a hospital to a primary care provider or from an inpatient to an outpatient setting. Effective care coordination reduces the risk of medical errors, enhances patient satisfaction, and improves health outcomes. Inadequate continuity of care, on the other hand, can lead to fragmented care, duplicative tests, and potentially harmful gaps in treatment.^[9,10]^ The clinician’s duty of care also encompasses patient advocacy. Clinicians are often uniquely positioned to advocate for their patients, addressing barriers to care and ensuring that patients receive the necessary services and support. This advocacy can involve helping patients navigate complex healthcare systems, securing access to necessary treatments, and supporting patients in making informed decisions. Clinicians may also advocate for broader systemic changes, such as improvements in healthcare policy, resource allocation, and public health initiatives. Advocacy efforts should be patient-centered, focusing on the patient’s best interests while respecting their autonomy and values. At times, this advocacy may involve challenging institutional policies or norms that are not in the best interest of patients, requiring clinicians to balance their professional responsibilities with their duty to the healthcare institution.^[11,12]^ The duty of care also involves managing the clinician’s health and well-being. Burnout, stress, and mental health issues are prevalent among healthcare professionals, often stemming from the demanding nature of the job, long hours, and the emotional toll of caring for patients. Clinicians are responsible for managing their well-being, as their physical and mental health directly impacts their ability to provide quality care. This self-care includes seeking support when needed, maintaining a healthy work-life balance, and engaging in activities that promote physical and emotional well-being. Healthcare institutions also support clinician well-being, provide resources, foster a supportive work environment, and address systemic factors contributing to clinician burnout. A healthy and well-supported clinician workforce is essential for ensuring patient safety and delivering high-quality care.^[13,14]^ Moreover, the duty of care requires clinicians to improve quality continuously. This involves regularly evaluating and reflecting on their practice, identifying areas for improvement, and implementing changes to enhance patient care. Continuous quality improvement can take many forms, such as participating in clinical audits, engaging in research and evidence-based practice, and adopting new technologies or treatment modalities. Clinicians must also stay informed about emerging health threats, such as new diseases or public health crises, and be prepared to adapt their practice accordingly. Quality improvement is not a one-time effort but an ongoing commitment to excellence in patient care.^[15,16]^

## 12. Legal consequences

In the healthcare sector, legal consequences arise from various actions or omissions by healthcare professionals, institutions, and regulatory bodies. These consequences are governed by a complex interplay of statutory law, case law, and professional regulations to ensure patients’ safety, rights, and well-being. Legal ramifications can result from breaches of duty, negligence, malpractice, violations of patient rights, and noncompliance with regulatory standards. Medical malpractice is one of the most prominent areas where legal consequences manifest in healthcare.^[10]^ It occurs when a healthcare provider deviates from the accepted standard of care, harming a patient. Malpractice claims typically involve allegations of negligence, which is a failure to exercise the care that a reasonably prudent healthcare provider would in similar circumstances. To establish a medical malpractice case, a plaintiff must demonstrate 4 elements: duty, breach, causation, and damages. The duty refers to the legal obligation of the healthcare provider to provide care; the breach is the violation of this duty through an act or omission; causation links the breach to the harm suffered; and damages refer to the actual harm or loss incurred by the patient. Legal consequences of malpractice can include civil liability, where the healthcare provider may be required to compensate the patient financially. In severe cases, criminal charges may be brought if the provider’s actions are deemed reckless or intentional, leading to criminal penalties such as fines or imprisonment.^[11,21]^ Informed consent is another critical area with significant legal implications. Informed consent is a legal and ethical requirement that obligates healthcare providers to disclose sufficient information about a proposed treatment or procedure, including its risks, benefits, and alternatives, allowing patients to make informed decisions about their care. Failure to obtain informed consent can result in legal consequences, such as civil lawsuits for battery (unlawful touching) or negligence. In these cases, patients may claim they were not adequately informed and thus could not provide valid consent. The consequences for healthcare providers and institutions can include financial compensation to the patient and reputational damage. Furthermore, the lack of proper documentation of informed consent can weaken the provider’s defense in legal proceedings, emphasizing the importance of thorough and accurate medical record-keeping.^[13,41]^ Patient confidentiality is another domain with substantial legal consequences. The obligation to maintain the confidentiality of patient information is enshrined in laws such as the HIPAA in the United States and the GDPR in Europe. These regulations set stringent standards for protecting personal health information, including requirements for secure storage, transmission, and access controls. Breaches of confidentiality, whether intentional or accidental, can lead to legal actions against healthcare providers and institutions. These actions may result in civil penalties, including fines and compensation for damages caused by the breach. In some cases, regulatory bodies may impose sanctions, such as revoking licenses or certifications, particularly if the breach involves gross negligence or is part of a pattern of noncompliance. Additionally, breaches of confidentiality can lead to criminal charges if they involve fraudulent activities, such as identity theft or unauthorized disclosure for financial gain.^[16,51]^ Regulatory compliance is another critical aspect with legal consequences. Healthcare providers and institutions must adhere to various regulations and standards set by governmental and professional bodies. These regulations cover various areas, including clinical practice guidelines, patient safety protocols, billing and coding standards, and healthcare facility operations. Noncompliance can result in legal actions, such as fines, sanctions, or exclusion from government healthcare programs like Medicare and Medicaid. For instance, fraudulent billing practices, such as upcoding (charging for more expensive services than those provided) or billing for services not rendered, can lead to significant financial penalties and criminal charges under laws like the False Claims Act in the United States. Regulatory bodies may also conduct audits and investigations, leading to further legal consequences if violations are discovered.^[8,17]^ The legal consequences of medical errors are another area of concern. Medical errors, defined as preventable adverse events or actions that lead to patient harm, can have significant legal ramifications for healthcare providers and institutions. These errors can occur in various forms, including diagnostic, medication, surgical, and patient management errors. The legal consequences of medical errors often overlap with those of medical malpractice, as they may involve allegations of negligence or breach of duty. In addition to civil liability, healthcare providers may face disciplinary actions from professional licensing boards, including suspension or revocation of their medical licenses. Institutions may also face regulatory penalties, increased scrutiny, and loss of accreditation. Moreover, medical errors can lead to loss of patient trust and damage the institution’s reputation, which can have long-term financial and operational impacts.^[10,19]^ The doctrine of vicarious liability further complicates the legal landscape for healthcare institutions. Under this doctrine, an employer (such as a hospital or clinic) can be held liable for the negligent acts of its employees if those acts occur within the scope of employment. This means that hospitals can be sued for the actions of their doctors, nurses, and other staff, even if the institution itself did not directly commit the negligent act. The legal consequences for the institution can include financial compensation to the patient and increased insurance premiums. Hospitals and clinics, therefore, have a vested interest in ensuring that their staff are adequately trained, competent, and adhere to established protocols and standards of care. They must also implement comprehensive risk management strategies to mitigate potential legal liabilities.^[11,12]^ Another significant area with legal consequences is the duty to warn. This legal principle requires healthcare providers to disclose certain information to third parties when there is a foreseeable risk of harm. For example, mental health professionals may have a duty to warn potential victims or law enforcement if a patient expresses a credible threat of violence. Similarly, healthcare providers may be required to report communicable diseases to public health authorities to prevent the spreading of infections. Failure to fulfill this duty can result in legal liability for any harm resulting from nondisclosure. However, this duty must be balanced with maintaining patient confidentiality, making it a complex legal and ethical issue.^[13,14]^ The legal consequences of end-of-life decisions are also notable. Healthcare providers often face complex legal and ethical issues related to decisions about life-sustaining treatments, do-not-resuscitate (DNR) orders, and advance directives. Failure to follow a patient’s advance directive or the wishes of their legal representative can lead to legal actions for battery or negligence. Conversely, providing treatment against a patient’s wishes can also result in legal liability. These situations often require careful documentation and consultation with legal and ethical experts to navigate the complex interplay of laws, regulations, and ethical principles. Additionally, healthcare providers must be aware of the legal requirements in their jurisdiction regarding end-of-life care, as these can vary widely and significantly impact legal liability.^[15,16]^ In healthcare research, legal consequences can arise from ethical violations, such as conducting studies without informed consent, failing to protect participant confidentiality, or not adhering to approved study protocols. These violations can result in legal actions, including civil lawsuits, regulatory penalties, and loss of research funding. Researchers and institutions may also face sanctions from institutional review boards or ethics committees, which oversee the ethical conduct of research. The legal implications extend to the publication of research findings, where misconduct, such as data fabrication or plagiarism, can lead to retractions, legal actions, and damage to professional reputations. The complex legal and ethical landscape of healthcare research underscores the importance of rigorous adherence to ethical standards and regulatory requirements.^[17,18]^ The intersection of healthcare and technology introduces additional legal challenges. With the increasing use of EHRs, telemedicine, and digital health tools, legal consequences can arise from data breaches, cybersecurity incidents, and the misuse of technology. For example, a data breach that exposes patient information can result in legal actions under data protection laws, such as HIPAA or GDPR. Telemedicine practices also raise legal issues related to licensure, jurisdiction, and standard of care. Providers must ensure they are licensed to practice in the state or country where the patient is located and adhere to the applicable standards of care. Failure to do so can result in legal actions, including fines, loss of licensure, and criminal charges. Rapid healthcare technology advancement requires continuous legal and regulatory adaptation to address emerging challenges and protect patient rights.^[19,20]^

## 13. Data breach notification requirements

Data breach notification requirements are critical regulatory measures designed to protect individuals’ personal information and ensure that organizations handle data responsibly. These requirements mandate that organizations notify affected individuals and, in some cases, regulatory authorities and other entities when a data breach occurs. A data breach can involve unauthorized access, acquisition, disclosure, or loss of sensitive information, such as personal data, financial records, or health information. These notification requirements provide transparency, enable affected individuals to take protective actions, and hold organizations accountable for data security practices.^[21–23]^ One of the most prominent legal frameworks governing data breach notification is the GDPR in the European Union. The GDPR, which came into effect in May 2018, sets stringent data protection and privacy requirements, including specific provisions for data breach notification. Under the GDPR, organizations must notify the relevant data protection authority within 72 hours of becoming aware of a data breach unless the breach is unlikely to risk individuals’ rights and freedoms. Additionally, if the breach poses a high risk to individuals’ rights and freedoms, the organization must notify the affected individuals without delay. The notification to the data protection authority must include details about the nature of the breach, the categories and approximate number of affected individuals and data records, the consequences of the breach, and the measures taken or proposed to address the breach and mitigate its effects.^[1,2]^ The GDPR’s data breach notification requirements also emphasize transparency and accountability. Organizations must provide clear and plain language explanations in their notifications, enabling affected individuals to understand the nature of the breach and the potential impact on their data. Furthermore, organizations must maintain records of all data breaches, regardless of whether notification is required, as part of their accountability obligations under the GDPR. Failure to comply with the GDPR’s notification requirements can result in substantial fines, up to 20 million euros or 4% of the organization’s total global turnover, whichever is higher. These stringent penalties underscore the importance of compliance and the potential financial consequences for organizations that fail to meet their obligations.^[3,4]^ Data breach notification laws vary by state in the United States, creating a complex legal landscape for organizations operating across multiple jurisdictions. All 50 states, the District of Columbia, and several U.S. territories have enacted data breach notification laws. While these laws share common elements, such as the requirement to notify affected individuals and, in some cases, regulatory authorities, they differ in terms of the definition of a data breach, the types of data covered, the timing and content of notifications, and exemptions. For example, California’s data breach notification law, the California Consumer Privacy Act (CCPA), requires businesses to notify affected California residents if their personal information is compromised. The CCPA also mandates that businesses provide a general description of the incident, the types of personal information involved, and contact information for further inquiries. The CCPA and other state laws impose civil penalties for noncompliance, adding another layer of legal risk for organizations.^[5,6]^ Another significant data breach notification framework is the HIPAA in the United States, specifically the HIPAA Breach Notification Rule. This rule applies to covered entities, such as healthcare providers, health plans, healthcare clearinghouses, and business associates. Under HIPAA, covered entities must notify affected individuals, the U.S. Department of HHS, and, in some cases, the media of unsecured PHI breaches. Notification to individuals must occur without unreasonable delay and no later than 60 days after the breach’s discovery. The notification must include a description of the breach, the types of PHI involved, the steps individuals should take to protect themselves, and the measures taken by the covered entity to investigate the breach and prevent future occurrences. The HIPAA Breach Notification Rule also requires covered entities to report breaches affecting 500 or more individuals to the HHS, which then posts the information on its public breach portal, often referred to as the “wall of shame.”^[7,8]^ The obligation to notify affected individuals and regulatory authorities is not limited to the GDPR, U.S. state laws, and HIPAA. Many other countries have enacted data breach notification laws, reflecting the global trend toward stronger data protection regulations. For instance, Australia’s NDB scheme, part of the Privacy Act 1988, requires organizations to notify the OAIC and affected individuals of eligible data breaches. An eligible data breach occurs when personal information is accessed or disclosed without authorization, which is likely to seriously harm any of the individuals to whom the information relates. The notification must include the organization’s contact details, a description of the breach, the types of information involved, and recommendations for affected individuals to protect themselves. The NDB scheme emphasizes prompt and transparent communication with the regulator and affected individuals.^[9,10]^ Compliance with data breach notification requirements presents several challenges for organizations. One of the primary challenges is the need to identify and assess a data breach quickly. Organizations must have robust incident detection and response mechanisms to detect breaches promptly, determine their scope and impact, and assess whether notification is required. This process can be complex, especially in cases involving sophisticated cyberattacks or when third-party service providers hold the breached data. Organizations must also navigate the complexities of different legal requirements across jurisdictions, particularly if they operate internationally. This includes understanding each jurisdiction’s specific notification thresholds, timelines, and content requirements, which can vary significantly. Additionally, organizations must balance the need for transparency with the risk of causing unnecessary panic or harm to affected individuals, particularly if the full extent of the breach is not yet known.^[11,12]^ Another challenge in compliance is the potential reputational damage and loss of customer trust resulting from a data breach. Even if an organization complies with all legal notification requirements, the public disclosure of a breach can harm its reputation and erode customer confidence. Customers may fear that their data is insecure, leading to a loss of business and a decrease in market value. Organizations must, therefore, manage their communications carefully, providing clear and accurate information to affected individuals while demonstrating a commitment to addressing the breach and improving security measures. This includes offering support services, such as credit monitoring or identity theft protection, to help affected individuals mitigate the potential consequences of the breach.^[13,14]^ Data breach notification requirements also highlight the importance of preventive measures and data security best practices. Organizations are encouraged to implement strong data protection measures, including encryption, access controls, and regular security audits, to reduce the risk of data breaches. Additionally, having a well-defined incident response plan is crucial for ensuring a swift and effective response to data breaches. Such plans should outline the roles and responsibilities of key personnel, the steps for identifying and containing the breach, the process for assessing the breach’s impact, and the procedures for notifying affected individuals and regulatory authorities. Regular training and drills can help ensure staff are prepared to respond to a breach effectively and comply with legal requirements.^[15,16]^

## 14. Best practices for clinicians

Evidence-based medicine (EBM) is a cornerstone of best practices in clinical care. EBM involves integrating the best available research evidence with clinical expertise and patient values to make informed decisions about patient care. This approach requires clinicians to stay updated with the latest research findings, guidelines, and clinical trials. By applying evidence-based guidelines, clinicians can provide scientifically validated treatments, improving the effectiveness and efficiency of care.^[16,17]^ For example, using standardized treatment protocols for conditions such as hypertension, diabetes, and cancer has been shown to improve patient outcomes and reduce variations in care. Moreover, EBM encourages using clinical decision-making tools and algorithms, which help assess risk, choose appropriate diagnostic tests, and select the best treatment options. This systematic approach minimizes the reliance on anecdotal evidence or outdated practices, leading to more consistent and predictable patient outcomes.^[1,2]^ Patient-centered care is another critical aspect of best practices. This approach emphasizes the importance of understanding and respecting patients’ preferences, needs, and values. It involves actively engaging patients in their care, providing clear and comprehensive information, and supporting them in making informed decisions. Effective communication is a key component of patient-centered care. Clinicians should use plain language, avoid medical jargon, and ensure patients fully understand their diagnosis, treatment options, and the potential risks and benefits. Additionally, clinicians should be attentive to patients’ cultural, social, and emotional needs, providing empathetic and compassionate care. This approach improves patient satisfaction and adherence to treatment plans and fosters a stronger therapeutic relationship, which is crucial for successful outcomes. In practice, patient-centered care can include shared decision-making, personalized care plans, and the involvement of family members or caregivers in the treatment process.^[3,4]^ Interdisciplinary collaboration is essential for delivering comprehensive and coordinated care. Modern healthcare often involves a team of professionals with diverse expertise, including physicians, nurses, pharmacists, therapists, social workers, and other specialists. Effective interdisciplinary collaboration requires clear communication, mutual respect, and a shared commitment to patient welfare. Clinicians should actively participate in multidisciplinary team meetings, case discussions, and care planning sessions. This collaborative approach integrates different perspectives and expertise, leading to more holistic and effective treatment plans. For example, in managing chronic conditions such as heart failure or diabetes, a team-based approach can address care’s medical, psychological, and social aspects, ensuring that all patient needs are met. Additionally, interdisciplinary collaboration can help prevent medical errors, streamline care transitions, and reduce hospital readmissions.^[5,6]^ Continuous professional development (CPD) is a vital component of best practices for clinicians. The medical field constantly evolves, with new research, technologies, and treatment modalities emerging regularly. Clinicians must engage in lifelong learning to stay current with these advancements and maintain their clinical competence. CPD can take various forms, including attending conferences, participating in workshops, completing online courses, and reading medical journals. Many professional organizations and regulatory bodies require clinicians to earn continuing education credits for their licensure and certification requirements. Furthermore, clinicians should seek opportunities for skill enhancement, such as simulation training, peer review, and mentoring. By actively pursuing CPD, clinicians can enhance their knowledge, improve their clinical skills, and provide their patients with the highest standard of care.^[7,8]^ Ethical considerations are fundamental to best practices in clinical care. Clinicians are entrusted with significant responsibilities and must adhere to ethical principles such as autonomy, beneficence, non-maleficence, and justice. Respecting patient autonomy involves recognizing patients’ rights to make their own decisions about their care, including the right to refuse treatment. Clinicians should provide patients with all necessary information, support their decision-making process, and respect their choices, even if they differ from the clinician’s recommendations. Beneficence requires clinicians to act in the patient’s best interest, providing beneficial treatments and avoiding those that may cause harm. Non-maleficence, closely related to beneficence, emphasizes the obligation to do no harm. Clinicians should carefully consider any intervention’s potential risks and benefits and strive to minimize harm. Justice in healthcare involves treating all patients fairly and equitably, ensuring that resources are allocated appropriately, and avoiding discrimination based on race, gender, socioeconomic status, or disability.^[9,10]^ Documentation is another critical aspect of best practices for clinicians. Accurate and thorough documentation of patient encounters, treatment plans, and clinical decisions is essential for ensuring continuity of care, legal protection, and quality assurance. Clinicians should record relevant patient information, including history, examination findings, diagnostic tests, treatment decisions, and follow-up plans, in a timely and clear manner. Documentation should be objective, avoiding subjective opinions or unnecessary commentary. In addition to traditional paper records, many healthcare systems now use EHRs, which offer advantages such as improved accessibility, data security, and integration with other clinical systems. Proper documentation also facilitates communication among healthcare providers, allowing seamless care transitions and reducing the risk of medical errors.^[11,12]^ Infection control and prevention are crucial elements of best practices, particularly in hospital and clinical settings. Healthcare-associated infections pose significant risks to patients and can lead to serious complications, prolonged hospital stays, and increased healthcare costs. Clinicians must adhere to established infection control protocols, such as hand hygiene, personal protective equipment, and sterilization of medical instruments. In infectious disease outbreaks or pandemics, clinicians should follow public health guidelines and recommendations, including vaccination, screening, and isolation procedures. Infection control practices also involve educating patients and their families about infection prevention measures, such as handwashing and the appropriate use of antibiotics. By maintaining high infection control standards, clinicians can protect patients, staff, and the wider community from infectious diseases.^[13,14]^ Patient safety is a paramount concern in clinical practice. Clinicians must identify and mitigate potential safety risks, such as medication errors, diagnostic errors, and procedural complications. Implementing safety protocols, such as standardized checklists, double-checking medication dosages, and conducting thorough preoperative assessments, can help reduce the likelihood of adverse events. Clinicians should also encourage a culture of safety within their organizations, where staff feel comfortable reporting near misses and adverse events without fear of retribution. Root cause analysis of incidents can provide valuable insights into system failures and inform strategies for preventing future occurrences. Patient safety initiatives, such as adopting safety bundles and using simulation training, are effective tools for improving safety outcomes and fostering a culture of continuous improvement.^[15,16]^ Cultural competence is an increasingly important aspect of best practices, especially in diverse and multicultural societies. Clinicians must be sensitive to their patients’ cultural beliefs, values, and practices, as these can significantly influence health behaviors and treatment preferences. Cultural competence involves understanding the cultural context of health and illness, recognizing potential language barriers, and providing culturally appropriate care. Clinicians should strive to communicate effectively with patients from diverse backgrounds, using interpreters or translation services when necessary. Additionally, they should respect cultural differences in health practices and collaborate with patients to develop treatment plans that align with their cultural values. Culturally competent care not only improves patient satisfaction and adherence to treatment but also helps reduce health disparities and promote health equity.^[17,18]^ Finally, using technology in healthcare is an essential component of best practices. Technological advancements, such as telemedicine, mobile health apps, and electronic health records, have transformed how clinicians deliver care. Telemedicine, for instance, allows for remote consultations, expanding access to care for patients in rural or underserved areas. Mobile health apps can provide patients with tools for managing chronic conditions, tracking medications, and monitoring vital signs. Electronic health records facilitate the seamless sharing of patient information among healthcare providers, improving care coordination and reducing the risk of errors. Clinicians should embrace these technologies, ensuring they are used effectively and ethically. This includes understanding technology’s limitations and potential risks, such as data privacy concerns, and ensuring that digital tools are integrated into clinical practice to enhance patient care.^[19,20]^

## 15. Insurance and risk management in healthcare cybersecurity

Due to its valuable data, the healthcare industry has become a prime target for cybercriminals. Medical records contain sensitive personal information, including Social Security numbers, medical histories, and financial data, making them highly valuable on the black market. Cyberattacks on healthcare organizations can take various forms, including data breaches, ransomware attacks, and phishing schemes.^[45–47]^ The consequences of such incidents can be severe, ranging from financial penalties and lawsuits to reputational damage and loss of patient trust. In response to these threats, healthcare organizations have increasingly turned to cyber insurance to protect themselves from the financial impact of cyber incidents. Cyber insurance is a specialized insurance product designed to cover losses and liabilities arising from cyber incidents. It typically covers a range of costs associated with data breaches, including legal fees, notification costs, public relations expenses, and the cost of investigating and responding to an incident. Cyber insurance policies may sometimes cover the costs of restoring lost or damaged data and compensating for business interruption losses. The coverage provided by cyber insurance can vary widely depending on the policy, the insurer, and the healthcare organization’s specific needs. Therefore, it is crucial for healthcare organizations to carefully evaluate their risk profile and select a policy that provides adequate coverage.^[1,2]^ The process of obtaining cyber insurance begins with a thorough risk assessment. This involves identifying and evaluating the organization’s cybersecurity risks, including various cyber incidents’ likelihood and potential impact. Risk assessment typically includes an analysis of the organization’s IT infrastructure, data storage practices, access controls, and incident response capabilities. Additionally, insurers may consider factors such as the organization’s size, the types of data it handles, and its history of cyber incidents. Insurers will determine the policy’s appropriate coverage limits and premiums based on the risk assessment results. Healthcare organizations must be transparent and thorough in this process, as failing to disclose relevant information can result in denied claims or insufficient coverage.^[3,4]^ In addition to obtaining cyber insurance, healthcare organizations must implement robust risk management strategies to mitigate the likelihood and impact of cyber incidents. Risk management involves proactively identifying, assessing, and mitigating cybersecurity risks. This includes implementing technical controls, such as firewalls, encryption, and multi-factor authentication, as well as administrative controls, such as policies and procedures for data handling and incident response. Training and awareness programs are also critical components of risk management, as human error is common in cybersecurity incidents. By educating staff on cybersecurity best practices and how to recognize phishing attempts and other threats, healthcare organizations can significantly reduce their risk exposure.^[5,6]^ One of the key challenges in healthcare cybersecurity is balancing security measures with the need for accessibility and usability. Healthcare providers require access to patient data to deliver timely and effective care, and overly restrictive security measures can impede this access. Therefore, risk management strategies must be tailored to the specific needs of the healthcare environment. For example, while multi-factor authentication is a recommended security measure, it may not be practical in all clinical settings. In such cases, alternative measures, such as monitoring and auditing access logs, can provide additional security without compromising patient care. It is also essential for healthcare organizations to regularly review and update their security measures to keep pace with evolving threats and technological advancements.^[7,8]^ Incident response planning is another critical aspect of risk management in healthcare cybersecurity. Despite the best efforts to prevent cyber incidents, breaches occur. An effective incident response plan outlines the steps the organization will take in the event of a cyber incident, including the roles and responsibilities of staff, communication protocols, and procedures for containing and mitigating the impact of the incident. Key elements of an incident response plan include identifying and isolating affected systems, preserving evidence for investigation, notifying affected individuals and regulatory authorities, and restoring normal operations. Healthcare organizations should regularly test and update their incident response plans to ensure they are prepared to respond swiftly and effectively to a cyber incident.^[9,10]^ The role of cyber insurance in incident response cannot be understated. In the aftermath of a cyber incident, healthcare organizations may face significant costs, including legal fees, fines, and the cost of notifying affected patients. Cyber insurance can provide financial support for these expenses, helping organizations manage the financial impact of the incident. Additionally, many cyber insurance policies offer access to a network of experts, including legal counsel, public relations specialists, and forensic investigators, who can assist in responding to the incident. This support can be invaluable in managing a cyber incident’s complex and often overwhelming aftermath.^[11,12]^ The regulatory landscape also plays a significant role in shaping healthcare organizations’ risk management and insurance strategies. Regulations such as the HIPAA in the United States, the GDPR in the European Union, and other national and international data protection laws impose strict requirements on the handling and protecting patient data. Noncompliance with these regulations can result in substantial fines, penalties, and legal and reputational consequences. Therefore, healthcare organizations must ensure that their cybersecurity practices, including cyber insurance and risk management strategies, align with regulatory requirements. This includes conducting regular audits and assessments to ensure compliance and address identified gaps.^[13,14]^ Another important consideration in healthcare cybersecurity is the potential impact of emerging technologies. AI, ML, and the IoT significantly benefit patient care and operational efficiency. However, they also introduce new cybersecurity risks. For example, AI systems may be vulnerable to attacks that manipulate their algorithms, leading to incorrect diagnoses or treatment recommendations. IoT devices, such as connected medical devices, can be exploited as entry points for cyberattacks if not properly secured. Healthcare organizations must consider these emerging risks in their risk management strategies and ensure that their cyber insurance policies cover incidents involving new technologies.^[15,16]^

## 16. Collaborative approach

The principles underlying a collaborative approach include mutual respect, shared goals, open communication, and collective decision-making. Mutual respect is foundational, ensuring that all participants value each other’s contributions and perspectives. Doctors, nurses, and allied health professionals have an equal voice in patient care discussions in a healthcare setting. Shared goals align all parties’ efforts, ensuring everyone works towards a common objective.^[48,49]^ This alignment is crucial in interdisciplinary research, where scientists from different fields must converge on a unified research question or outcome. Open communication is essential for transparency and trust, allowing team members to share information freely and address issues as they arise. Finally, collective decision-making fosters a sense of ownership and accountability among participants, as decisions are made through consensus rather than unilateral directives.^[1,2]^ One of the primary benefits of a collaborative approach is the pooling of knowledge and expertise. For instance, an interdisciplinary team comprising physicians, nurses, pharmacists, social workers, and other specialists can provide comprehensive patient care. Each member brings a unique perspective and skill set, enabling the team to address the multifaceted needs of patients. This holistic approach is particularly beneficial in managing complex chronic conditions, where medical, psychological, and social factors must be considered. Similarly, in research and development, collaboration between experts from different disciplines can lead to innovative solutions that may not have emerged from a single-disciplinary perspective. The convergence of diverse ideas often sparks creativity and leads to breakthroughs that advance knowledge and practice.^[3,4]^ Efficiency is another significant advantage of collaboration. By working together, teams can divide tasks according to expertise, streamlining processes and reducing redundancy. In a corporate setting, for instance, a collaborative approach can expedite project timelines by allowing simultaneous work on different aspects of a project. This division of labor speeds up completion and improves the output’s quality, as the most qualified individuals handle each component. In public policy, collaboration between government agencies, nongovernmental organizations, and private entities can lead to more effective and efficient implementation of programs. For example, public-private partnerships in infrastructure development can leverage the strengths of both sectors, combining public oversight and accountability with private sector efficiency and innovation.^[5,6]^ The collaborative approach also fosters learning and professional development. Team members can broaden their knowledge and skills by engaging with individuals from diverse backgrounds and expertise. In healthcare education, interprofessional training programs expose students to different roles and responsibilities within the healthcare system, promoting a more integrated understanding of patient care. Such exposure prepares future professionals to work effectively in interdisciplinary teams. In corporate environments, cross-functional teams enable employees to gain insights into different business functions, enhancing their versatility and career development. This continuous learning environment is essential for adapting to the rapidly changing demands of modern professions and industries.^[7,8]^ Despite its numerous benefits, a collaborative approach is not without challenges. One of the main obstacles is the potential for conflicts arising from differing perspectives, priorities, and communication styles. In healthcare, for instance, doctors and nurses may have different approaches to patient care, leading to disagreements. Similarly, scientists may have conflicting methodologies or theoretical frameworks in cross-disciplinary research. Effective collaboration requires mechanisms for conflict resolution, such as open dialogue, mediation, and negotiation. Establishing clear roles and responsibilities can also help minimize conflicts by delineating the scope of each participant’s contributions. Another challenge is coordinating efforts, especially in large and diverse teams. Effective collaboration necessitates strong leadership and project management skills to ensure that all activities are well-coordinated and aligned with the shared goals.^[9,10]^ Trust is another critical factor in successful collaboration. Trust among team members fosters open communication, reduces anxiety, and promotes a willingness to share ideas and take risks. Building trust requires time and consistent effort, including demonstrating reliability, competence, and integrity. In healthcare teams, trust is particularly crucial as it affects patient outcomes. Patients are more likely to receive safe and effective care when healthcare professionals trust one another and communicate openly. In research, trust enables sharing sensitive data and intellectual property, facilitating joint ventures and collaborative studies. Establishing trust can be challenging, especially in newly formed teams or partnerships, but it is essential for long-term collaboration success.^[11,12]^ Another significant challenge is the alignment of organizational cultures and objectives. In public-private collaborations, for instance, government entities and private companies may have different priorities and operational cultures. Governments often prioritize public welfare and compliance with regulations, while private companies may focus on profitability and innovation. Bridging these cultural and objective differences requires negotiation and establishing shared values and goals. A clear and transparent governance structure can help manage these differences, ensuring all parties work towards a common purpose. In healthcare, aligning the cultures of different professional groups, such as physicians, nurses, and administrators, is crucial for cohesive and efficient patient care delivery.^[13,14]^ Technology is vital in facilitating collaboration, especially in today’s digital age. Communication and collaboration tools, such as video conferencing, project management software, and collaborative platforms, enable teams to work together seamlessly, regardless of geographical location. These tools are particularly useful in international collaborations, allowing for real-time communication and data sharing across borders. In research, digital platforms facilitate the collaborative analysis of large datasets and coauthoring scientific papers. However, the reliance on technology also poses challenges, such as data security and the need for technological literacy among all team members. Ensuring all participants have access to the necessary tools and training is essential for effective collaboration.^[15,16]^ The importance of a collaborative approach has been particularly evident during the COVID-19 pandemic. The global health crisis required unprecedented collaboration across countries, sectors, and disciplines. Governments, international organizations, pharmaceutical companies, and healthcare providers had to work together to develop vaccines, share information, and coordinate responses. This collaboration was crucial in accelerating vaccine development, managing healthcare resources, and implementing public health measures. The pandemic highlighted the importance of global solidarity and cooperation in addressing complex and widespread challenges. It also underscored the need for robust systems and structures to facilitate collaboration in crisis.^[17,18]^ Collaboration between educators, students, and parents is essential for effective learning in the educational sector. Teachers collaborate to design curricula, share best practices, and develop teaching strategies. Collaboration with students involves engaging them in learning, encouraging active participation, and providing feedback. Parents are also important collaborators, supporting their children’s education at home and communicating with teachers. In higher education, interdisciplinary programs and research projects provide students with opportunities to collaborate across disciplines, preparing them for the complexities of the professional world. Collaboration in education enhances learning outcomes and fosters a sense of community and shared responsibility among all stakeholders.^[19,20]^

## 17. Case study: cybersecurity breach in a healthcare setting

This information is based on an original case observed and managed by the authors at our clinic involving a significant cybersecurity breach. The incident highlighted the vulnerabilities inherent in healthcare information systems and the critical importance of robust cybersecurity measures and proactive management to protect sensitive patient data. The breach occurred within our hospital’s EHR system, the primary repository for patient information, including personal identifiers, medical histories, treatment plans, and billing information. The system had been operational for several years, with regular updates and patches applied per standard protocols. Despite these measures, a sophisticated cyber-attack successfully infiltrated our network, raising concerns about the effectiveness of our existing security framework. The breach began with a phishing attack targeting hospital staff. An email, disguised as an internal communication, contained a link to a malicious website designed to capture login credentials. The email from the IT department appeared, creating a sense of urgency regarding a supposed system update. Several staff members, including those with administrative access, fell victim to this deception, unknowingly providing their login information to the attackers. Once inside the system, the attackers moved laterally across our network, escalating their privileges to gain access to sensitive areas of the EHR system. They employed various techniques, including installing keyloggers and exploiting vulnerabilities in unpatched software components. The attackers’ primary objective was to exfiltrate patient data, including PHI and financial records. The breach was first detected when our cybersecurity monitoring tools noticed unusual network activity. The system logs indicated unauthorized access to multiple accounts and the extraction of large volumes of data. An internal investigation was initiated involving our IT and cybersecurity teams, who worked to contain the breach and assess the damage. Their efforts were complemented by external cybersecurity consultants who provided additional expertise and resources. During the initial response phase, the hospital’s IT team isolated affected systems to prevent further unauthorized access and initiated a forensic analysis to determine the scope of the breach. The analysis revealed that the attackers had accessed and potentially exfiltrated a substantial amount of patient data, including sensitive information such as social security numbers, medical records, and insurance details. Following the containment and analysis phases, the hospital immediately notified affected individuals as required by applicable data breach notification regulations. Affected patients were informed of the breach through direct communication, including email and postal notifications, detailing the nature of the incident, the potential risks, and the steps to mitigate the impact. Additionally, the hospital offered free credit monitoring services to affected individuals to help them manage any potential financial risks resulting from the breach. The breach also necessitated a review of the hospital’s cybersecurity policies and practices. An audit revealed several areas needing improvement, including outdated software, inadequate staff training on recognizing phishing attempts, and insufficient MFA protocols. In response, the hospital undertook a comprehensive overhaul of its cybersecurity infrastructure. This included implementing advanced threat detection systems, enhancing staff training programs, and enforcing stricter access controls and encryption standards. The incident prompted a broader discussion within the hospital and the wider healthcare community about the importance of cybersecurity in protecting patient data. The breach was a stark reminder of the evolving nature of cyber threats and the necessity for continuous vigilance and adaptation to new risks. It also highlighted the critical need for healthcare organizations to invest in robust cybersecurity measures and to foster a culture of security awareness among staff members. The hospital faced several legal and regulatory challenges after the breach. An investigation was conducted by regulatory bodies to determine compliance with data protection laws and to assess the adequacy of the hospital’s response. The hospital cooperated fully with these investigations, providing detailed accounts of the incident and the steps to address the vulnerabilities. This cooperation was crucial in mitigating potential legal repercussions and maintaining trust with patients and regulatory authorities. The breach also had significant financial implications for the hospital. The costs associated with the incident included expenses for cybersecurity consultants, legal fees, notification and credit monitoring services, and potential fines for noncompliance with data protection regulations. Additionally, the incident affected the hospital’s reputation, leading to increased scrutiny from patients and stakeholders regarding the security of their personal information. To address these challenges, the hospital implemented measures to enhance its overall cybersecurity posture. These included the development of a comprehensive incident response plan, regular security training for staff, and ongoing investments in advanced security technologies. The hospital also collaborated with other healthcare organizations and cybersecurity experts to share knowledge and best practices for managing cyber threats. The breach has had lasting effects on the hospital’s approach to cybersecurity. It underscored the need for continuous improvement and adaptation in response to the ever-changing threat landscape. The hospital’s experience has also contributed to the broader discourse on healthcare cybersecurity, providing valuable insights and lessons for other organizations facing similar challenges.

## 18. Impact on patient care and trust in healthcare cybersecurity

One of the most direct impacts of cybersecurity breaches in healthcare is the potential disruption of medical services. Hospitals and clinics rely heavily on digital systems for patient record management, diagnostic imaging, communication, and treatment planning. A cyber attack compromising these systems can lead to care delays, medical record errors, and the inability to access critical patient information.^[10]^ For example, ransomware attacks, where cybercriminals encrypt data and demand a ransom for its release, can lock healthcare providers out of their systems. Such incidents can force providers to resort to manual operations, significantly slowing down processes and increasing the likelihood of errors. These disruptions can be life-threatening, particularly in cases where timely access to medical records and diagnostic information is crucial for patient survival.^[15,52]^ Data breaches, where unauthorized individuals access sensitive patient information, pose another significant threat. Such breaches can expose PHI, including medical histories, test results, and treatment plans. The exposure of this data can have far-reaching consequences for patients, including identity theft, discrimination, and stigmatization. For instance, patients with sensitive health conditions may face discrimination if their information is publicly disclosed or falls into the wrong hands. Furthermore, the knowledge that their private medical data could be exposed can deter patients from seeking care, especially for stigmatized conditions such as mental health issues or sexually transmitted infections. This chilling effect on patient disclosure can lead to underreporting of symptoms and conditions, resulting in inadequate treatment and poorer health outcomes.^[53,54]^ The psychological impact of cybersecurity incidents on patients cannot be underestimated. Patients who learn that their personal information has been compromised may experience anxiety, stress, and a loss of trust in the healthcare system. This psychological burden can be exacerbated by the fear of financial losses or misuse of their data. The erosion of trust is particularly concerning, as trust is a foundational element of the patient-provider relationship. Patients trust healthcare providers with their most sensitive information, which is crucial for effective communication, accurate diagnosis, and adherence to treatment plans. This trust can be severely damaged when cybersecurity incidents occur, making patients less likely to share critical information or follow medical advice.^[6,55]^ Moreover, the impact of cybersecurity incidents extends beyond individual patients to affect the healthcare system as a whole. Healthcare providers may face significant financial costs related to incident response, legal liabilities, and fines from regulatory bodies. These financial burdens can strain healthcare organizations, particularly smaller clinics, and hospitals, potentially leading to cutbacks in services or even closure. Additionally, the reputational damage resulting from a cybersecurity breach can have long-term consequences. Healthcare organizations that experience breaches may find it challenging to regain the trust of their patients and the public, leading to a decline in patient volume and revenue. The loss of patient trust can also hinder collaborative efforts in healthcare, as patients may be reluctant to participate in research studies or share their data for public health initiatives.^[8,57]^ The ethical implications of cybersecurity in healthcare are also significant. Healthcare providers have an ethical duty to protect patient information and ensure the confidentiality and integrity of data. Failure to do so violates legal obligations and ethical principles such as autonomy, beneficence, and non-maleficence. Autonomy involves respecting patients’ rights to control their personal information, while beneficence and non-maleficence require providers to act in patients’ best interests and prevent harm. When cybersecurity incidents occur, these ethical principles are compromised, potentially causing harm to patients and undermining the ethical foundation of healthcare practice.^[9,10]^ Healthcare providers can take several steps to mitigate the impact of cybersecurity incidents on patient care and trust. First, implementing robust cybersecurity measures is essential. This includes using strong encryption methods, regularly updating software, employing multi-factor authentication, and conducting regular security audits. Additionally, healthcare organizations should have comprehensive incident response plans in place. These plans should outline procedures for detecting, responding to, and recovering from cybersecurity incidents and communicating with patients and stakeholders. Effective communication is crucial in maintaining patient trust, particularly in the aftermath of a breach. Transparent and timely communication can help reassure patients that their providers are taking the necessary steps to protect their information and prevent future incidents.^[11,12]^ Education and training are also critical components of a comprehensive cybersecurity strategy. Healthcare providers, including clinicians, administrative staff, and IT personnel, must be educated about the risks associated with cybersecurity and trained in best practices for data protection. This training should include recognizing phishing attempts, securing mobile devices, and safeguarding sensitive information. By fostering a culture of cybersecurity awareness, healthcare organizations can reduce the risk of incidents and enhance their ability to respond effectively when they occur.^[13,14]^ Moreover, engaging patients in cybersecurity efforts can also be beneficial. Educating patients about the importance of cybersecurity and how they can protect their information can empower them to safeguard their data actively. For instance, patients should be encouraged to use secure communication channels when interacting with healthcare providers and to be cautious about sharing personal information online. Additionally, healthcare organizations can provide resources and support to help patients monitor their financial and health records for signs of misuse or fraud.^[15,16]^ Finally, collaboration among healthcare providers, government agencies, and cybersecurity experts is crucial for addressing the complex challenges of cybersecurity in healthcare. Governments can play a vital role by establishing and enforcing regulations that set standards for data protection in healthcare. These regulations can help ensure that all healthcare organizations, regardless of size, adhere to best practices for cybersecurity. Collaboration with cybersecurity experts can also provide healthcare organizations with the expertise and tools to defend against sophisticated cyber threats. Public–private partnerships can also facilitate the sharing of information about emerging threats and best practices, helping to strengthen the overall cybersecurity posture of the healthcare sector.^[17,18]^

## 19. Patient rights and advocacy in the context of cybersecurity

The core of patient rights revolves around privacy, confidentiality, and informed consent. These rights are enshrined in various legal and ethical frameworks, including national laws, international agreements, and professional codes of conduct. These rights are critically important in cybersecurity because they underpin patients’ trust in healthcare providers to protect their personal and medical information.^[53–55]^ A breach of this trust, whether through unauthorized access, data breaches, or misuse of information, can have profound implications for patients, including emotional distress, identity theft, and financial loss. Privacy is a fundamental right that refers to an individual’s control over their personal information. In the healthcare setting, this includes the right to have one’s medical records and personal health information kept confidential and used only for legitimate purposes. The confidentiality of patient information is protected by various laws and regulations, such as the HIPAA in the United States, the GDPR in the European Union, and other national and international data protection laws.^[1,2]^ These regulations establish standards for handling, storing, and sharing patient data, ensuring it is protected from unauthorized access and misuse. Informed consent is another critical component of patient rights. It requires that patients are fully informed about the nature and purpose of any medical treatment or procedure, including how their data will be used and protected. This also extends to using electronic health records and other digital tools in the digital era. Patients must be aware of how their data is collected, stored, and shared, and they should have the opportunity to consent to or opt out of certain data uses.^[3,4]^ Ensuring that patients understand their rights and the implications of their consent is essential for maintaining trust and upholding ethical standards in healthcare. The role of advocacy is crucial in safeguarding patient rights in the realm of cybersecurity. Patient advocacy involves representing and defending the interests of patients, particularly in situations where their rights may be at risk. In cybersecurity, advocacy efforts ensure that healthcare organizations and policymakers prioritize protecting patient information and address any vulnerabilities that may arise from digital health technologies. Patient advocacy organizations are key in raising awareness about cybersecurity risks and advocating for stronger protections. These organizations educate patients about their rights and the importance of data security and influence policy and regulatory developments. For example, organizations such as the Patient Privacy Rights Foundation and the Electronic Frontier Foundation advocate for policies and practices that enhance the security and privacy of patient data.^[5,6]^ They also provide resources and support for patients affected by data breaches or other cybersecurity incidents, helping them navigate the legal and practical aspects of addressing these issues. Healthcare organizations are also responsible for advocating for patient rights by implementing robust cybersecurity measures and maintaining transparency about their data protection practices. This includes ensuring that patients are informed about how their data is used, obtaining explicit consent for data sharing, and providing clear information on reporting any concerns or breaches.^[7,8]^ By adopting best practices in cybersecurity and prioritizing patient privacy, healthcare organizations can demonstrate their commitment to protecting patient rights and fostering trust. Policymakers play a critical role in shaping the legal and regulatory frameworks that govern the protection of patient data. Effective policies and regulations are essential for establishing standards and guidelines for data security and ensuring that healthcare organizations comply with these standards. Policymakers must consider the evolving landscape of digital health technologies and the associated risks to patient privacy when developing and updating regulations.^[9,10]^ Collaboration between policymakers, healthcare providers, and patient advocacy groups is essential for creating comprehensive and effective cybersecurity policies that address the needs and concerns of all stakeholders. One of the key challenges in the realm of patient rights and cybersecurity is balancing the need for data security with the need for accessibility and usability. While strong cybersecurity measures are necessary to protect patient information, they must not impede care delivery or create unnecessary patient barriers. For example, overly restrictive access controls or complex authentication processes can hinder healthcare providers’ ability to promptly access and share patient information. Therefore, it is important to design cybersecurity measures that balance security and functionality, ensuring that patient data is protected without compromising the quality of care.^[11,12]^ In addition to legal and regulatory considerations, ethical issues play a significant role in patient rights and cybersecurity. Ethical principles such as beneficence, non-maleficence, and justice guide healthcare practice and inform decisions about data protection. Healthcare providers must ensure that their cybersecurity practices align with these principles, protecting patient information while minimizing potential harm and ensuring equitable access to care.^[13,14]^ Ethical considerations also extend to emerging technologies, such as AI and ML, which can raise questions about data privacy and the potential for bias in decision-making. The impact of data breaches on patient trust cannot be overstated. When patients learn that their personal or medical information has been compromised, it can lead to losing confidence in their healthcare providers and the healthcare system. Rebuilding trust after a breach requires a transparent and accountable response, including timely notification of affected individuals, clear communication about the steps to address the breach and support for those affected.^[15,16]^ Healthcare organizations must also learn from breaches to improve cybersecurity and prevent future incidents. There are several areas where further research and action are needed to strengthen protecting patient rights in cybersecurity. Research is needed to explore the effectiveness of existing regulations and policies in addressing cybersecurity risks and to identify best practices for balancing security and accessibility. Additionally, there is a need for continued advocacy efforts to raise awareness about patient rights and to influence policy and regulatory developments. Healthcare organizations must also invest in ongoing education and training for staff to ensure they are equipped to handle cybersecurity challenges and uphold patient rights.^[56–58]^

## 20. The role of professional organizations and guidelines in healthcare cybersecurity

Professional organizations in healthcare, such as the American Medical Association (AMA), the American Nurses Association, and the Health Information and Management Systems Society (HIMSS), have been instrumental in advocating for the integration of strong cybersecurity measures. These organizations recognize the importance of protecting patient data as a core component of patient care.^[59,60]^ They have developed various guidelines and resources to assist healthcare providers in enhancing their cybersecurity posture. One of the primary contributions of these organizations is the development of guidelines that set minimum standards for cybersecurity practices. These guidelines often cover many topics, including data encryption, access control, incident response, and employee training.^[61,62]^ Guidelines provided by professional organizations serve as a benchmark for healthcare institutions, ensuring a consistent approach to cybersecurity across the industry. By adhering to these guidelines, healthcare providers can demonstrate their commitment to protecting patient data and comply with regulatory requirements. For example, the AMA has published guidelines emphasizing securing EHRs through encryption and access controls. These guidelines are crucial for preventing unauthorized access to patient information, thus maintaining the confidentiality and integrity of data. Similarly, HIMSS has developed a comprehensive cybersecurity framework that outlines best practices for managing cybersecurity risks, including conducting regular risk assessments and implementing multi-factor authentication.^[63,64]^ In addition to providing guidelines, professional organizations play a vital role in policy advocacy. They work closely with government agencies, such as the Department of HHS and the Office for Civil Rights, to influence the development of policies and regulations that govern healthcare cybersecurity. These collaborations ensure that the regulations are practical and applicable to the realities of healthcare practice. For instance, professional organizations’ input has significantly shaped the HIPAA Security Rule, which sets national standards for protecting electronic health information. By participating in policy development, these organizations help ensure that the regulations are stringent enough to protect patient data and feasible for healthcare providers to implement.^[65,66]^ Moreover, professional organizations often act as a bridge between healthcare providers and cybersecurity experts. They facilitate knowledge exchange and provide a platform for sharing best practices and lessons learned from real-world experiences. Conferences, webinars, and publications are some mediums through which these organizations disseminate information. For example, HIMSS hosts an annual conference that brings healthcare professionals, IT experts, and policymakers to discuss the latest trends and challenges in healthcare cybersecurity. These events are invaluable for healthcare providers, offering them insights into emerging threats and innovative solutions. By staying informed about the latest developments in cybersecurity, healthcare professionals can better protect their systems and patient data.^[67,68]^ Education and training are other critical areas where professional organizations significantly impact. Recognizing that healthcare providers may not always have the technical expertise required to manage cybersecurity threats, these organizations offer training programs and certification courses. For instance, the HIMSS Certified in Healthcare Privacy and Security (CHPS) credential is a recognized certification that equips healthcare professionals with the knowledge and skills needed to manage healthcare data privacy and security. Similarly, the International Association of Privacy Professionals (IAPP) offers privacy laws and data protection certifications. These educational initiatives are crucial for building a workforce knowledgeable about cybersecurity and capable of implementing best practices.^[69,70]^ The role of guidelines and professional organizations extends to developing and enforcing ethical standards in healthcare cybersecurity. These standards ensure that healthcare providers respect patient rights and maintain trust. For instance, the AMA’s Code of Medical Ethics includes provisions related to the ethical management of patient information, emphasizing the importance of confidentiality and the responsible use of technology. By adhering to these ethical standards, healthcare providers can navigate the ethical dilemmas that arise in the digital age, such as balancing patient privacy with the need for data sharing in public health initiatives. Ethical guidelines also help providers manage situations where patient data may be compromised, ensuring they act transparently and accountable.^[11,12]^ Another crucial aspect of a professional organization’s role is incident response and crisis management. In a cybersecurity incident, such as a data breach or ransomware attack, healthcare providers must swiftly mitigate the damage and protect patient data. Professional organizations provide guidelines and resources to help healthcare providers develop and implement effective incident response plans. These plans typically include identifying and containing the threat, notifying affected individuals, and complying with legal and regulatory requirements. For instance, the NIST has developed a Cybersecurity Framework that includes a section on incident response, offering detailed guidance on managing and recovering from cybersecurity incidents. By following these guidelines, healthcare providers can minimize the impact of incidents and restore normal operations more quickly.^[13,14]^ In addition to responding to incidents, professional organizations emphasize the importance of resilience and recovery. This includes restoring systems and data and learning from incidents to prevent future occurrences. Professional organizations often conduct post-incident analyses and publish reports highlighting lessons learned and best practices for improving cybersecurity resilience. These resources are invaluable for healthcare providers, offering them insights into the latest threats and vulnerabilities and helping them strengthen their cybersecurity defenses. By promoting a continuous improvement and learning culture, professional organizations help healthcare providers stay ahead of evolving cyber threats.^[15,16]^ The global nature of cyber threats necessitates international collaboration, and professional organizations play a key role in fostering such cooperation. Cybersecurity is a borderless issue, and healthcare providers worldwide face similar challenges. Professional organizations often collaborate with international bodies, such as the World Health Organization and the International Telecommunication Union, to develop global standards and share best practices. These collaborations are essential for addressing the global nature of cyber threats and ensuring a coordinated response. For instance, the Global Digital Health Partnership, an international collaboration of governments, healthcare providers, and professional organizations, works to advance digital health and cybersecurity practices worldwide. By participating in such initiatives, professional organizations contribute to the global effort to enhance healthcare cybersecurity.^[17,18]^

## 21. Emerging technologies and international perspectives on cybersecurity in healthcare

AI has revolutionized numerous aspects of healthcare, including diagnostics, treatment planning, and patient monitoring. AI algorithms, particularly those based on ML and deep learning, have demonstrated exceptional capabilities in analyzing vast amounts of medical data, identifying patterns, and making predictions. For example, AI systems are increasingly used for analyzing medical imaging, such as detecting anomalies in radiology scans with high accuracy.^[1]^ Similarly, AI-driven predictive analytics can forecast patient outcomes, aiding in early intervention and personalized treatment plans.^[2]^ However, the deployment of AI in healthcare introduces significant cybersecurity risks. AI systems are vulnerable to various attacks, including adversarial attacks, where malicious inputs are designed to deceive the AI algorithms and alter their predictions or decisions. For instance, researchers have demonstrated how slight perturbations in medical images can mislead AI-based diagnostic tools, potentially leading to incorrect diagnoses.^[3]^ Additionally, AI systems often require access to large datasets, including sensitive patient information. Ensuring the security and privacy of these datasets is crucial to prevent unauthorized access and data breaches. Recent studies have highlighted the need for robust security measures to protect AI systems in healthcare. One study emphasizes the importance of securing the data pipelines to train AI models and implementing strong authentication mechanisms to prevent unauthorized modifications.^[4]^ Another study suggests integrating AI-specific security measures, such as anomaly detection algorithms, that can identify unusual patterns indicative of potential attacks.^[5]^ These measures are essential to safeguard AI systems from malicious threats and ensure their reliability and effectiveness in healthcare applications. Quantum computing represents another groundbreaking technology with the potential to impact cybersecurity in healthcare. Unlike classical computers, which use bits to represent data as either 0 or 1, quantum computers use quantum bits or qubits that can exist in multiple states simultaneously. This property allows quantum computers to perform complex calculations at unprecedented speeds, potentially solving problems that are infeasible for classical computers.^[6]^ In the context of cybersecurity, quantum computing poses both opportunities and threats. One of the most significant concerns is quantum computers’ ability to break existing cryptographic algorithms. Many current encryption methods, such as RSA and elliptic curve cryptography (ECC), rely on the difficulty of certain mathematical problems, like factoring large numbers or solving discrete logarithms, which are computationally intensive for classical computers. However, quantum computers can solve these problems more efficiently using algorithms like Shor’s, rendering existing cryptographic protocols vulnerable.^[7]^ This vulnerability could compromise the security of encrypted health data, necessitating the development of quantum-resistant encryption methods. On the other hand, quantum computing also offers potential solutions to cybersecurity challenges. QKD is a technique that uses the principles of quantum mechanics to create secure communication channels. QKD allows 2 parties to share encryption keys with a level of security theoretically immune to eavesdropping, as any attempt to intercept the key would disrupt the quantum state and be detectable.^[8]^ Implementing QKD in healthcare could enhance the security of sensitive patient information and protect against data breaches. International perspectives on cybersecurity regulations reflect the diverse approaches taken by different countries to address these emerging threats and challenges. The regulatory landscape varies significantly, with each jurisdiction implementing its frameworks and requirements for protecting healthcare data. In the United States, the HIPAA and the HITECH Act form the foundation of cybersecurity regulations in healthcare. HIPAA mandates stringent standards for safeguarding ePHI and requires healthcare organizations to implement administrative, physical, and technical safeguards.^[9]^ The HITECH Act further strengthens data protection by promoting the meaningful use of EHRs and introducing breach notification requirements.^[10]^ However, the U.S. regulatory framework primarily focuses on data protection and privacy, with less emphasis on addressing specific threats posed by emerging technologies such as AI and quantum computing. In contrast, the European Union’s GDPR provides a comprehensive data protection approach that includes specific health data provisions. GDPR emphasizes the principles of data protection by design and by default, requiring organizations to implement measures that ensure the security and privacy of personal data.^[11]^ The regulation also introduces the concept of data protection impact assessments for processing activities that pose high risks to individuals’ rights and freedoms.^[12]^ These provisions are relevant to emerging technologies like AI, requiring organizations to assess and mitigate potential risks associated with using AI systems in healthcare. Following Brexit, the United Kingdom has retained GDPR principles through the UK GDPR and the Data Protection Act 2018. The UK GDPR aligns closely with the EU GDPR, emphasizing the need for robust data protection measures and transparency in data processing activities.^[13]^ The UK’s approach also includes guidance on specific AI-related issues, such as the need for explainability and accountability in automated decision-making processes.^[14]^ Canada’s healthcare cybersecurity approach is governed by the PIPEDA and provincial PHIPAs. PIPEDA sets out principles for collecting, using, and disclosing personal information, including health data, and requires organizations to implement security safeguards.^[15]^ Provincial legislation, such as Ontario’s PHIPA, provides more detailed requirements for health information, including breach notification obligations.^[16]^ The Canadian regulatory framework emphasizes data protection and privacy but may need to adapt to address emerging AI and quantum computing threats. In Australia, the Privacy Act 1988 and the APPs govern handling personal information, including health data. The NDB scheme requires organizations to report certain types of data breaches to affected individuals and the OAIC.^[17]^ The Australian regulatory framework emphasizes data protection and breach notification but may need to address specific challenges related to emerging technologies. Japan’s APPI provides rules for handling personal information, including health data. The APPI requires organizations to implement measures to ensure data security and establish procedures for responding to data breaches.^[18]^ The PIPC oversees compliance and enforcement. Japan’s regulatory approach emphasizes data protection but may need to address emerging AI and quantum computing threats. China’s Cybersecurity Law and PIPL represent comprehensive data protection regulations focusing on critical information infrastructure, including healthcare systems. The Cybersecurity Law mandates data security measures for operators of critical infrastructure, while the PIPL regulates the processing of personal information and introduces detailed requirements for data protection.^[19]^ The Chinese regulatory framework emphasizes data protection and cybersecurity but may need to adapt to emerging technological challenges. In India, healthcare cybersecurity is governed by the Information Technology Act 2000 (IT Act) and the proposed PDPB. The IT Act includes provisions for cybersecurity and data protection, while the PDPB, once enacted, will introduce comprehensive data protection requirements.^[20]^ India’s regulatory framework focuses on data protection and cybersecurity but may need to address specific challenges emerging technologies pose.

## 22. AI in cybersecurity

AI’s application in cybersecurity primarily revolves around its ability to analyze vast amounts of data quickly and accurately. Traditional cybersecurity methods often struggle to keep pace with the increasing volume and sophistication of cyber threats. With its advanced algorithms and ML capabilities, AI provides a valuable tool for overcoming these limitations.^[10]^ Machine learning, a subset of AI, enables systems to learn from data and improve performance over time without explicit programming. This capability is particularly useful in identifying patterns and anomalies in network traffic, which can signal potential security breaches. One of the primary applications of AI in cybersecurity is in threat detection and response. AI-powered systems can analyze network traffic, user behavior, and other data sources to identify unusual activities that may indicate a cyber attack. For example, anomaly detection algorithms can flag deviations from normal patterns, such as unusual login times or unexpected data transfers, allowing security teams to investigate and respond rapidly to potential threats. Recent studies have demonstrated the effectiveness of AI in detecting zero-day attacks, which are previously unknown vulnerabilities that traditional security solutions might not recognize.^[11,12]^ AI also plays a crucial role in automating incident response. In the event of a detected threat, AI systems can take immediate actions to contain and mitigate the attack’s impact. This includes isolating affected systems, blocking malicious traffic, and applying patches to vulnerabilities. Automation helps to reduce the response time and minimize human error, which can be critical in preventing or limiting damage from cyber attacks. Research has shown that AI-driven automation can significantly enhance the efficiency and effectiveness of incident response efforts.^[4,31]^ In addition to threat detection and response, AI is used in predictive analytics to anticipate and prepare for potential cyber threats. AI systems can forecast future attack vectors and vulnerabilities by analyzing historical data and identifying trends. This predictive capability allows organizations to implement security measures and defenses proactively before an attack occurs. For example, AI can predict the likelihood of a targeted attack based on factors such as recent threat intelligence, network configurations, and known vulnerabilities.^[6,15]^ The integration of AI into cybersecurity strategies also brings several benefits. One of the most significant advantages is processing and analyzing large volumes of data in real time. Traditional security systems often struggle with the sheer amount of data generated by modern networks and applications. AI’s ability to handle big data and extract meaningful insights enables more effective monitoring and protection of digital assets. Furthermore, AI systems can continuously learn and adapt to new threats, improving their accuracy and effectiveness. However, the use of AI in cybersecurity also presents several challenges and concerns. One of the primary challenges is the risk of adversarial attacks, where malicious actors exploit weaknesses in AI algorithms to evade detection or manipulate system behavior. For example, attackers may use techniques such as data poisoning to corrupt the training data of ML models, leading to inaccurate or biased results.^[8,17]^ Ensuring the robustness and resilience of AI systems against such attacks is a critical area of ongoing research. Another challenge is the potential for false positives and false negatives. AI systems, while highly effective, are not infallible. False positives occur when legitimate activities are incorrectly flagged as threats, leading to unnecessary investigations and potential disruptions. False negatives, on the other hand, occur when the system does not detect actual threats. Balancing the sensitivity and specificity of AI algorithms is essential to minimize these issues and ensure accurate threat detection.^[9,10]^ Ethical considerations also play a significant role in deploying AI in cybersecurity. One major concern is privacy. AI systems often require access to large amounts of data, including personal and sensitive information, to function effectively. Ensuring that AI applications comply with data protection regulations and respect user privacy is crucial. Transparency in how AI systems collect, process, and use data can help build trust and address privacy concerns.^[11,12]^ Bias is another ethical issue associated with AI in cybersecurity. Machine learning algorithms can inadvertently perpetuate existing biases present in the training data. For example, suppose an AI system is trained on data that reflects historical biases. In that case, it may produce biased outcomes, such as disproportionately targeting certain user groups or failing to detect specific threats. Addressing bias in AI systems requires careful consideration of data sources, algorithm design, and ongoing monitoring to ensure fairness and accuracy.^[13,14]^ The legal and regulatory landscape surrounding AI in cybersecurity is also evolving. Various jurisdictions are developing frameworks and guidelines to govern AI technologies, including data protection laws, cybersecurity regulations, and AI transparency and accountability standards. Organizations must navigate these regulations to ensure compliance and mitigate legal risks. For example, the European Union’s GDPR includes provisions related to automated decision-making and the right to explanation, which are relevant to AI applications.^[15,16]^ Several case studies illustrate the successful application of AI in cybersecurity. For instance, a recent deployment of AI-driven threat detection systems in a large financial institution demonstrated significant improvements in detecting and responding to cyber threats. The system was able to identify previously unknown attack patterns and reduce response times, leading to enhanced overall security.^[17,18]^ Another example is the use of AI in securing healthcare systems, where AI algorithms have been employed to monitor network traffic and detect anomalies indicative of potential breaches, helping to protect sensitive patient data.^[19,20]^

## 23. Quantum computing and cybersecurity

Quantum computing represents a revolutionary shift in computational capabilities with profound implications for cybersecurity. Unlike classical computers, which use bits as the smallest unit of information (0 or 1), quantum computers use quantum bits or qubits. These qubits can represent and process a much larger range of values simultaneously due to the principles of superposition and entanglement.^[41]^ This unique capability allows quantum computers to solve certain problems significantly faster than classical computers, but it also introduces new challenges and risks for cybersecurity. One of the most notable implications of quantum computing for cybersecurity is its potential to undermine current cryptographic systems. Classical encryption algorithms, such as RSA (Rivest–Shamir–Adleman) and ECC, rely on the difficulty of solving certain mathematical problems. For instance, RSA’s security is based on the challenge of factoring large integers, while ECC relies on the difficulty of solving the discrete logarithm problem.^[42]^ These problems are computationally intensive for classical computers but become feasible for quantum computers due to their ability to perform parallel computations. Quantum computers can leverage algorithms such as Shor’s algorithm to solve these mathematical problems efficiently, breaking widely used cryptographic systems. Shor’s algorithm, developed by mathematician Peter Shor in 1994, allows a quantum computer to factor large integers in polynomial time, making it possible to decrypt data protected by RSA encryption.^[1]^ Similarly, Shor’s algorithm can be adapted to solve discrete logarithm problems, potentially compromising ECC-based encryption.^[2]^ The advent of quantum computing thus poses a significant threat to the confidentiality and integrity of encrypted data. Researchers are actively developing quantum-resistant cryptographic algorithms to address these challenges, also known as post-quantum cryptography. These algorithms are designed to be secure against both classical and quantum attacks. For example, lattice-based cryptography, hash-based cryptography, and code-based cryptography are being explored as potential solutions to replace existing cryptographic systems.^[3]^ Lattice-based cryptographic schemes, such as those based on the Learning With Errors problem, offer strong security guarantees and resist quantum attacks.^[4]^ Hash-based cryptographic schemes, like the Merkle tree, provide security based on hash functions rather than mathematical problems susceptible to quantum attacks.^[5]^ Another area of concern is QKD, a technique that uses quantum mechanics to exchange encryption keys securely. QKD relies on the principles of quantum mechanics to detect eavesdropping during the key exchange process. If an eavesdropper tries to intercept the key, the quantum state of the key is altered, alerting the communicating parties to the presence of an intruder.^[6]^ While QKD offers a theoretically secure method for key exchange, its practical implementation faces challenges related to the distance over which keys can be exchanged and the cost of quantum communication infrastructure.^[7]^ Despite these challenges, QKD represents a promising approach to enhancing cybersecurity in a post-quantum world. The potential impact of quantum computing extends beyond encryption. Quantum computers are also expected to influence data privacy and secure multi-party computations. For instance, quantum algorithms may facilitate more efficient data mining and pattern recognition, raising concerns about the privacy of sensitive information. Quantum computing could also affect secure multi-party computations, which enable parties to jointly compute a function without revealing their private inputs. Quantum advancements may alter the security guarantees of these computations, necessitating new protocols and approaches to ensure privacy and security.^[8]^ In addition to technical challenges, the rise of quantum computing introduces legal and regulatory considerations. As quantum computers become more capable, they may necessitate cybersecurity regulations and standards changes. For instance, data protection and encryption regulations may need to be updated to address the vulnerabilities introduced by quantum computing. Furthermore, organizations must stay informed about advancements in quantum technology and adopt quantum-resistant solutions to mitigate risks.^[9,10]^ Developing international standards for quantum-resistant cryptography and establishing guidelines for quantum-safe practices will be crucial for maintaining cybersecurity in the quantum era. Several initiatives are underway to prepare for the impact of quantum computing on cybersecurity. Governments, academic institutions, and private organizations are investing in research and development efforts to advance quantum-resistant technologies and frameworks. For example, the NIST is leading a project to standardize post-quantum cryptographic algorithms to guide organizations to transition to quantum-resistant systems.^[11]^ Additionally, collaborative efforts between researchers and industry stakeholders are focused on developing practical solutions and assessing the readiness of quantum technologies for real-world applications.^[12]^ Case studies highlight the practical implications of quantum computing for cybersecurity. For instance, a recent simulation study demonstrated how quantum algorithms could compromise existing encryption methods for securing financial transactions. The study emphasized the need for proactive measures to safeguard sensitive data against future quantum attacks.^[13]^ Another case involved assessing QKD systems in securing communication networks. The study revealed the potential benefits of QKD in enhancing the security of key exchanges but also noted the limitations regarding scalability and cost.^[14]^

## 24. Practical recommendations

Large hospitals and healthcare systems, with their extensive infrastructure and significant volumes of patient data, face unique cybersecurity challenges that require sophisticated and multi-layered approaches. One of the primary recommendations for these large institutions is implementing a comprehensive cybersecurity framework that includes advanced threat detection and response systems. This framework should encompass robust firewalls, IDS, and security information and event management systems to monitor and analyze network traffic for potential threats in real-time.^[1]^ Additionally, large hospitals should invest in endpoint protection solutions to secure medical devices and workstations against malware and other cyber threats.^[2]^ Given the complexity of their IT environments, large hospitals should also prioritize regular risk assessments and vulnerability scans to identify and address potential weaknesses in their systems. Third-party security experts should conduct these assessments to ensure an unbiased evaluation of the organization’s security posture.^[3]^ Furthermore, these institutions must establish a dedicated cybersecurity team responsible for managing and responding to security incidents and developing and implementing cybersecurity policies and procedures.^[4]^ Another crucial recommendation for large hospitals is to ensure comprehensive employee training and awareness programs. Staff members should be educated on cybersecurity best practices, including recognizing phishing attempts, using strong passwords, and following proper data handling procedures.^[5]^ Regular training sessions and simulated phishing exercises can help reinforce these practices and reduce the risk of human error, a common factor in many cybersecurity incidents.^[6]^ Medium-sized healthcare facilities, which often have fewer resources than large hospitals but still handle substantial amounts of patient data, require tailored cybersecurity measures that balance effectiveness with cost considerations. Implementing a robust cybersecurity framework similar to that large hospitals use is still important for these institutions, but it can be adjusted to fit their specific needs and budgets. Medium-sized facilities should focus on deploying essential cybersecurity tools, such as firewalls, anti-malware software, and encryption technologies, to protect sensitive data.^[7]^ Regular vulnerability assessments and penetration testing are also recommended for medium-sized facilities, although they can be conducted less frequently than in large hospitals. These assessments help identify and address potential security gaps and ensure that the organization’s cybersecurity measures remain effective against evolving threats.^[8]^ Medium-sized facilities should also establish clear incident response protocols and ensure that all staff members are familiar with these procedures to minimize the impact of any potential security breaches.^[9]^ Employee training and awareness programs are equally important for medium-sized facilities. Training should be tailored to address the organization’s specific cybersecurity risks and conducted regularly to keep staff updated on the latest threats and best practices.^[10]^ In addition, medium-sized facilities should consider implementing access controls and data encryption to protect sensitive information further and reduce the risk of unauthorized access.^[11]^ Small clinics, which often have limited resources and staff, face unique challenges in implementing comprehensive cybersecurity measures. However, several effective practices can still enhance their cybersecurity posture. For small clinics, focusing on fundamental cybersecurity practices is essential. This includes implementing strong password policies, using up-to-date anti-malware software, and regularly applying software updates and patches to address known vulnerabilities.^[12]^ Small clinics should also consider leveraging cloud-based cybersecurity solutions to provide robust protection without extensive on-premises infrastructure.^[13]^ These solutions often include data encryption, secure backup, and threat detection, which can help protect sensitive patient information from cyber threats. Additionally, small clinics should establish basic incident response protocols and ensure that staff members know the procedures to follow in case of a security breach.^[14]^ Employee training remains a critical component of cybersecurity for small clinics. Staff members should be educated on basic cybersecurity practices, such as recognizing phishing attempts and safeguarding patient data.^[15]^ Given the limited resources of small clinics, training programs can be streamlined and delivered through online modules or periodic workshops.^[16]^ Furthermore, small clinics should prioritize data backup and recovery processes to ensure that critical patient information can be restored during a cyber incident.^[17]^ Regardless of the size of the healthcare institution, it is crucial to recognize that cybersecurity is an ongoing process that requires continuous improvement and adaptation. Healthcare organizations must stay informed about emerging threats and evolving best practices to ensure their cybersecurity measures remain effective. Collaboration with industry partners, participation in cybersecurity forums and conferences, and engagement with cybersecurity experts can help organizations stay up-to-date with the latest developments and enhance their overall security posture.^[18]^

## 25. Ethical considerations in cybersecurity for clinicians

One of the cornerstone ethical principles in healthcare is patient confidentiality. Clinicians are ethically obligated to protect the privacy of patient information, a duty that has been magnified in the digital age. The digitization of health records and electronic health systems have made patient data more accessible and more vulnerable to breaches. Cybersecurity incidents like data breaches and hacking can compromise patient confidentiality, leading to unauthorized access to sensitive information. The ethical challenge for clinicians is to ensure that the systems they use are secure enough to protect against these threats. Failure to do so violates ethical principles and can erode patient trust, which is fundamental to the clinician-patient relationship.^[1,2]^ Informed consent is another critical ethical consideration. In cybersecurity, informed consent involves ensuring patients know how their data will be used, stored, and protected. This includes explaining the potential risks associated with data breaches and the measures in place to mitigate these risks. Clinicians must provide clear and comprehensive information, allowing patients to make informed decisions about their data. This ethical obligation is particularly challenging when dealing with technologies such as AI, where patients may not fully understand the complexities of data use and potential biases in algorithms.^[3,4]^ Ensuring transparency in how AI systems operate and the data they utilize is crucial for maintaining ethical standards in informed consent. The ethical use of AI in healthcare cybersecurity is an emerging concern. AI technologies offer significant potential in identifying and mitigating cyber threats through advanced analytics and ML algorithms. However, using AI also raises ethical questions about bias and fairness. For instance, AI systems trained on biased data may perpetuate inequalities, leading to discriminatory outcomes. Clinicians and healthcare organizations have an ethical responsibility to ensure that AI systems are transparent, fair, and free from biases. This involves scrutinizing the data used to train these systems and continuously monitoring their outputs for any signs of bias. The ethical implications of AI in cybersecurity extend beyond technical considerations to include broader societal impacts, such as the potential for AI to exacerbate disparities in healthcare access and quality.^[5,6]^ The principle of non-maleficence, or “do no harm,” is a foundational ethical guideline in healthcare. This principle requires clinicians to prevent harm caused by cybersecurity breaches. A failure to implement adequate cybersecurity measures can result in significant harm to patients, such as identity theft, unauthorized use of personal information, and disruptions to healthcare services. Clinicians must be proactive in ensuring that their systems are secure and follow best practices for cybersecurity, including regular software updates, strong passwords, and the implementation of multi-factor authentication. Additionally, clinicians must be prepared to respond swiftly and effectively to cybersecurity incidents to minimize harm.^[7,8]^ The ethical consideration of justice involves ensuring that all patients receive fair and equitable treatment. In cybersecurity, this principle relates to the equitable protection of all patient data, regardless of the patient’s socioeconomic status, geographic location, or other factors. Clinicians and healthcare organizations must strive to provide all patients with the same level of data protection. This is particularly important in a global context, where disparities in cybersecurity resources and capabilities can lead to unequal protection of patient data. For example, healthcare providers in low-resource settings may lack the financial or technical resources to implement robust cybersecurity measures, making them more vulnerable to attacks. Wealthier healthcare organizations and international bodies are ethically incumbent to support these providers in bolstering their cybersecurity defenses.^[9,10]^ Patient autonomy is also critical in the ethical landscape of healthcare cybersecurity. Autonomy refers to the right of patients to make informed decisions about their healthcare, including decisions related to the use and protection of their data. Clinicians must respect patients’ autonomy by providing the necessary information to make informed choices and honoring their preferences regarding data use. This includes respecting patients’ wishes to restrict access to certain information or to opt out of data sharing for research purposes. However, this ethical obligation must be balanced against the need to protect public health and safety, particularly when withholding information could pose a risk to others.^[11,12]^ Moreover, the ethical obligation of transparency extends to the aftermath of a cybersecurity incident. Clinicians and healthcare organizations must be transparent with patients about any breaches, including the nature of the breach, the data involved, and the steps to mitigate the impact. This transparency is essential for maintaining trust and allowing patients to take appropriate actions, such as monitoring their accounts for signs of identity theft. Ethical transparency also includes being honest about the limitations of current cybersecurity measures and the potential risks that patients face, even in the presence of robust security protocols.^[13,14]^ In addition to these ethical considerations, clinicians face the challenge of balancing ethical obligations with legal requirements. While laws and regulations provide a framework for protecting patient data, ethical considerations often require clinicians to go beyond mere compliance. For instance, legal standards may set minimum requirements for data protection, but ethical principles may demand more rigorous measures to ensure patient safety and privacy. Clinicians must navigate these overlapping spheres, making decisions that are not only legally compliant but also ethically sound. This often requires a nuanced understanding of legal obligations and ethical principles and a commitment to continuous learning and adaptation in the face of evolving cyber threats.^[15,16]^

## 26. International cooperation and legal harmonization in cybersecurity

The rapid advancement of digital technologies in healthcare has led to a proliferation of EHRs, telemedicine platforms, and other digital health tools. While these technologies have improved patient care and operational efficiency, they have also introduced new vulnerabilities that cybercriminals can exploit. Data breaches, ransomware attacks, and other cyber incidents pose significant risks to patient privacy, data integrity, and healthcare delivery.^[51,52]^ Given the cross-border nature of these threats, no single country can effectively tackle cybersecurity challenges in isolation. Therefore, international cooperation is essential for developing and implementing effective cybersecurity strategies. International cooperation in cybersecurity involves sharing information, expertise, and resources among countries to enhance collective security. This cooperation can take various forms, including bilateral and multilateral agreements, joint research initiatives, and collaborative response efforts. For example, organizations such as the International Telecommunication Union and the ISO play key roles in fostering international collaboration and developing standards for cybersecurity.^[53,54]^ These organizations facilitate the exchange of best practices, promote the adoption of international standards, and support capacity-building efforts in member countries. One of the primary challenges in achieving international cooperation is the lack of uniformity in cybersecurity regulations and practices. Countries have adopted varying cybersecurity approaches, influenced by their legal systems, cultural values, and technological infrastructures. For instance, the European Union’s GDPR sets stringent data protection and privacy requirements, including reporting data breaches within 72 hours.^[55]^ In contrast, the United States has a more fragmented regulatory landscape, with laws such as the HIPAA providing a framework for protecting patient information. Still, varying state laws complicate compliance.^[56]^ This regulatory divergence can create challenges for healthcare organizations that operate across multiple jurisdictions. Legal harmonization addresses these challenges by promoting consistency and alignment among national cybersecurity regulations. Harmonizing legal frameworks can help reduce regulatory burdens, streamline compliance processes, and enhance cross-border cooperation. For example, the European Union’s GDPR has influenced data protection regulations beyond Europe, with many countries adopting similar standards to facilitate international data flows and improve data protection.^[57]^ Similarly, developing international cybersecurity frameworks, such as the G20’s Framework for Cybersecurity and the Budapest Convention on Cybercrime, aims to establish common principles and practices for addressing cyber threats.^[8,9]^ Despite these efforts, significant challenges remain in achieving comprehensive legal harmonization. Differences in legal traditions, regulatory priorities, and enforcement mechanisms can hinder the development of a cohesive global framework for cybersecurity. Additionally, the rapid pace of technological innovation and the evolving nature of cyber threats require continuous updates to legal and regulatory frameworks, which can be difficult to achieve through international agreements alone.^[50,51]^ In addition to legal harmonization, international cooperation in cybersecurity involves collaborative efforts to strengthen global cybersecurity capabilities. This includes joint research and development initiatives, capacity-building programs, and information-sharing platforms. For example, the Global Forum on Cyber Expertise is an international initiative supporting cybersecurity capacity-building and knowledge-sharing through workshops, training, and collaborative projects.^[58]^ Similarly, the Cybersecurity and Infrastructure Security Agency in the United States collaborates with international partners to enhance global cybersecurity resilience through information sharing and joint exercises.^[59]^ Healthcare organizations also play a crucial role in international cooperation by adopting best practices, participating in global initiatives, and engaging with international standards organizations. For example, the HITRUST develops and maintains a common framework for managing cybersecurity and privacy risks in healthcare, which aligns with international standards and facilitates cross-border compliance.^[60]^ By adopting such frameworks and participating in global initiatives, healthcare organizations can enhance their cybersecurity posture and contribute to the collective effort to address cyber threats. Policymakers are critical in fostering international cooperation and legal harmonization in cybersecurity. They must collaborate with international partners to develop and implement common standards, share information and best practices, and address cross-border challenges. Policymakers should also support the development of international agreements and frameworks that promote consistency and alignment among national regulations. For example, the OECD’s Recommendation on Digital Security Risk Management for Economic and Social Prosperity provides guidelines for addressing digital security risks. It encourages member countries to adopt a risk-based approach to cybersecurity.^[15]^ Furthermore, policymakers should promote the inclusion of cybersecurity considerations in trade agreements and other international negotiations. The digital economy and global data flows are increasingly important components of international trade, and incorporating cybersecurity provisions into trade agreements can help address cross-border data protection and security issues.^[16]^ By integrating cybersecurity into trade agreements and other international frameworks, policymakers can facilitate the development of a cohesive global approach to cybersecurity and ensure that patient data is protected across borders. One of the key areas for future research is the development of global standards and best practices for cybersecurity in healthcare. Research should focus on identifying common challenges and solutions, evaluating the effectiveness of existing frameworks, and exploring new approaches to addressing emerging threats. Additionally, research should examine the impact of legal harmonization on cybersecurity outcomes and assess the effectiveness of international cooperation efforts in improving global cybersecurity resilience.^[17,18]^ Another important area for research is the role of emerging technologies in cybersecurity and their impact on legal and regulatory frameworks. Technologies such as AI and quantum computing have the potential to transform cybersecurity practices and pose new challenges for data protection. Research should explore the implications of these technologies for cybersecurity regulations and develop strategies for addressing their impact on patient privacy and data security.^[19,20]^

## 27. Conclusion

The legal landscape surrounding cybersecurity incidents in healthcare is complex and continually evolving. Clinicians are at the frontline of managing sensitive patient data; they bear significant legal responsibilities for protecting this information against cyber threats. The analysis highlights the critical role of cybersecurity measures in safeguarding patient data and ensuring compliance with regulatory frameworks. Effective implementation of these measures reduces the risk of data breaches and helps clinicians navigate the legal implications of cybersecurity incidents. Key findings include the recognition of the increasing reliance on advanced technologies, such as AI and quantum computing, which offer new opportunities and pose new challenges for cybersecurity in healthcare. While beneficial in detecting and responding to threats, AI introduces ethical and operational complexities that clinicians must address.

Similarly, quantum computing presents potential risks to traditional encryption methods, necessitating the development of new cryptographic strategies and legal standards. The comparative analysis of international regulations reveals that while some countries have robust frameworks for managing cybersecurity incidents, others must catch up, resulting in a fragmented global approach. This disparity underscores the need for harmonized international standards to ensure consistent patient data protection and streamline compliance for multinational healthcare organizations.

## 28. Future research directions

One crucial area for future investigation is the ethical implications of AI in healthcare. While AI has shown promise in enhancing cybersecurity through advanced threat detection and response, it raises significant concerns about bias and transparency. Future research should explore how biases in AI algorithms can impact patient outcomes and decision-making processes and develop frameworks to ensure that AI systems operate fairly and accountable.^[61–63]^ This research should also address how clinicians can manage these ethical challenges while leveraging AI technologies effectively. Quantum computing represents another frontier that warrants extensive research. The potential of quantum computing to undermine current encryption methods poses a substantial risk to data security in healthcare. Future studies should focus on developing and evaluating new cryptographic techniques capable of resisting quantum attacks. Additionally, there is a need to explore how legal frameworks can adapt to address the implications of quantum computing on data protection and cybersecurity standards.^[64]^

International regulatory harmonization is another critical area requiring further research. The comparative analysis of national regulations highlights significant disparities in how countries handle cybersecurity incidents. Future research should identify best practices and propose models for creating a cohesive international regulatory framework.^[65]^ This would facilitate consistent protection of patient data across borders and streamline compliance for global healthcare organizations. The effectiveness of current incident response protocols and their impact on legal liability for clinicians also need further examination. Research should evaluate how various response strategies influence legal outcomes and develop best practices for minimizing legal risks associated with data breaches.^[66,67]^ This includes investigating the role of incident response plans in reducing the likelihood of legal penalties and protecting professional reputations. Training and education for clinicians represent another crucial area for research. The effectiveness of cybersecurity training programs in enhancing clinicians’ ability to prevent and respond to cyber incidents should be thoroughly evaluated. Studies should explore the most effective methods for delivering this education and assess how it impacts clinicians’ preparedness and response capabilities. Finally, the challenges faced by small healthcare providers in implementing cybersecurity measures require focused research. Small clinics and practices often need more resources, making it challenging to adopt comprehensive cybersecurity strategies. Future research should identify cost-effective solutions and tools that small healthcare providers can use to improve their cybersecurity posture without straining their financial resources.^[68–71]^

## 29. Call to action

Clinicians are urged to engage in cybersecurity training and awareness programs to enhance their ability to identify and mitigate potential threats. Implementing best practices, such as regular software updates, secure communication channels, and stringent data encryption, is essential to protecting sensitive patient information. Additionally, clinicians should familiarize themselves with their legal responsibilities and the specific regulatory requirements for their jurisdiction. Healthcare organizations must prioritize the development of comprehensive cybersecurity policies and incident response plans tailored to their unique needs. Investing in advanced security technologies and fostering a culture of cybersecurity awareness can significantly reduce the risk of data breaches and ensure swift, effective responses to incidents. Ongoing collaboration between healthcare providers, policymakers, and cybersecurity experts is crucial to address the gaps identified in this research. Advocacy for stronger international regulations and standardized practices can help harmonize global efforts to protect patient data. Future research should continue to explore emerging technologies, ethical considerations, and practical solutions to enhance cybersecurity measures and mitigate legal risks.

## Acknowledgments

The authors would like to express gratitude to all individuals and institutions that contributed to the completion of this paper. Their support, guidance, and encouragement throughout the research process are deeply appreciated.

## Author contributions

**Conceptualization:** Chukwuka Elendu.

**Data curation:** Chukwuka Elendu.

**Formal analysis:** Chukwuka Elendu.

**Funding acquisition:** Chukwuka Elendu.

**Investigation:** Chukwuka Elendu.

**Methodology:** Chukwuka Elendu.

**Project administration:** Chukwuka Elendu, Eunice K. Omeludike, Praise O. Oloyede, Babajide T. Obidigbo, Janet C. Omeludike.

**Resources:** Chukwuka Elendu, Eunice K. Omeludike, Praise O. Oloyede, Babajide T. Obidigbo, Janet C. Omeludike.

**Software:** Chukwuka Elendu, Eunice K. Omeludike, Praise O. Oloyede, Babajide T. Obidigbo, Janet C. Omeludike.

**Supervision:** Chukwuka Elendu, Eunice K. Omeludike, Praise O. Oloyede, Babajide T. Obidigbo, Janet C. Omeludike.

**Validation:** Chukwuka Elendu, Eunice K. Omeludike, Praise O. Oloyede, Babajide T. Obidigbo, Janet C. Omeludike.

**Visualization:** Chukwuka Elendu, Eunice K. Omeludike, Praise O. Oloyede, Babajide T. Obidigbo, Janet C. Omeludike.

**Writing – original draft:** Chukwuka Elendu, Eunice K. Omeludike, Praise O. Oloyede, Babajide T. Obidigbo, Janet C. Omeludike.

**Writing – review & editing:** Chukwuka Elendu, Eunice K. Omeludike, Praise O. Oloyede, Babajide T. Obidigbo, Janet C. Omeludike.
